# Tandemly repeated DNA families in the mouse genome

**DOI:** 10.1186/1471-2164-12-531

**Published:** 2011-10-28

**Authors:** Aleksey S Komissarov, Ekaterina V Gavrilova, Sergey Ju Demin, Alexander M Ishov, Olga I Podgornaya

**Affiliations:** 1 Institute of Cytology RAS, 4 Tikhoretsky avenue, 194064, St. Petersburg, Russia; 2 Faculty of Biology and Soil Sciences, St. Petersburg State University, Universitetskaya nab. 7/9, St. Petersburg 199034, Russia; 3 Department of Anatomy and Cell Biology, University of Florida College of Medicine, 1376 Mowry, Gainesville FL 32610-3633, USA

## Abstract

**Background:**

Functional and morphological studies of tandem DNA repeats, that combine high portion of most genomes, are mostly limited due to the incomplete characterization of these genome elements. We report here a genome wide analysis of the large tandem repeats (TR) found in the mouse genome assemblies.

**Results:**

Using a bioinformatics approach, we identified large TR with array size more than 3 kb in two mouse whole genome shotgun (WGS) assemblies. Large TR were classified based on sequence similarity, chromosome position, monomer length, array variability, and GC content; we identified four superfamilies, eight families, and 62 subfamilies - including 60 not previously described. 1) The superfamily of centromeric minor satellite is only found in the unassembled part of the reference genome. 2) The pericentromeric major satellite is the most abundant superfamily and reveals high order repeat structure. 3) Transposable elements related superfamily contains two families. 4) The superfamily of heterogeneous tandem repeats includes four families. One family is found only in the WGS, while two families represent tandem repeats with either single or multi locus location. Despite multi locus location, TRPC-21A-MM is placed into a separated family due to its abundance, strictly pericentromeric location, and resemblance to big human satellites.

To confirm our data, we next performed *in situ *hybridization with three repeats from distinct families. TRPC-21A-MM probe hybridized to chromosomes 3 and 17, multi locus TR-22A-MM probe hybridized to ten chromosomes, and single locus TR-54B-MM probe hybridized with the long loops that emerge from chromosome ends. In addition to *in silico *predicted several extra-chromosomes were positive for TR by *in situ *analysis, potentially indicating inaccurate genome assembly of the heterochromatic genome regions.

**Conclusions:**

Chromosome-specific TR had been predicted for mouse but no reliable cytogenetic probes were available before. We report new analysis that identified *in silico *and confirmed *in situ *3/17 chromosome-specific probe TRPC-21-MM. Thus, the new classification had proven to be useful tool for continuation of genome study, while annotated TR can be the valuable source of cytogenetic probes for chromosome recognition.

## Background

Tandemly repeated DNA represents a significant portion of the mouse genome and include centromere and pericentromere regions. Although historically referred to as "junk DNA", Tandem Repeats (TR) appear to provide unique structural and functional characteristics due to their tandem organization. Tandemly repeated DNA contains multiple copies of a repeat unit (or monomer) arranged in a head to tail fashion. Centromeres from fission yeast to humans contain TR, and pericentromeric regions enriched in TR appearing to be critically important for establishing heterochromatin formation and proper chromosome segregation [1]. Some of these functions appear to involve RNA interference-mediated chromatin modifications [2-4].

TR content is well investigated in the human genome, and it shows a wide range of repeat sizes and organization, ranging from microsatellites of a few base pairs to megasatellites of up to several kilobases. Microsatellites and Variable Number Tandem Repeats (minisatellites or VNTRs) can be highly polymorphic and thus are used as genetic markers [5,6].

The centromeric region of human chromosomes contains alpha satellite DNA (satDNA), the largest TR family in the human genome. This family has been extensively studied and provides a paradigm for understanding the genomic organization of TR [7,8]. These tandem arrays are composed of either diverged monomers, with no higher order repeat structure, or as chromosome-specific Higher-Order Repeat (HOR) units characterized by distinct periodicity and arrangements of an integral number of basic monomers [9]. The HOR structure of human centromeric alpha satellite is important for centromere function [7].

In humans, the pericentromeric regions consist of alpha satDNA arrays that are surrounded by arrays of "classical" satellites (e.g. human satDNA 1-4) [10-13]. These pericentromeric regions have a specific high-order chromatin structure and might be responsible for chromatin spatial organization.

In the house mouse, *Mus musculus*, centromeric and pericentromeric regions are represented by two highly conserved, tandemly repeated sequences known as minor and major satellites (MiSat and MaSat, respectively, SATMIN and GSAT_MM in Repbase nomenclature). MiSat are composed of 120-bp AT-rich monomers that occupy 300-600 kb of the terminal region of all mouse telocentric (single-armed) chromosomes; these TR serve as the site of kinetochore formation and spindle microtubule attachment [14-18]. MaSat is more abundant and are combined from 234-bp monomers that resides adjacent to MiSat. MaSat are implicated into heterochromatin formation and sister chromatid cohesion [17,19]. Neither of these satDNA were identified at the centromere of the morphologically distinct acrocentric Y chromosome, which has a very short arm that distinguishes it from the telocentric autosomes and chromosome X [20]. Recently, the centromere of Y chromosome was shown to contain a highly diverged MiSat-like sequence (designated Ymin) with HOR organization previously not described for mouse MiSat arrays [20].

Here we report the analysis of mouse large TR genome organization by a combined bioinformatics and cytological approaches. All large TR found in two mouse whole genome shotgun assemblies (WGS) were classified into four superfamilies, eight families, and 62 subfamilies, including 60 not described yet. The proposed classification is based on array similarity, monomer length, the degree of unit similarity, position on the reference genome chromosome assemblies, and GC content. Three TR were selected for the experimental work due to their abundance in the WGS. All array-based probes recognize chromosomes predicted *in silico*.

The data reported here represent the overall genome wide assessment of the number, position and organization of large TR in the sequenced mouse genome. Annotated TR could be an important resource for further characterization and overall understanding of the mouse genome.

## Results

### Tandemly repeated DNA in mouse whole genome shotgun assemblies

For the initial search of large TR we used two WGS assemblies: Mouse Genome Sequencing Consortium (MGSC) and Celera assemblies [21,22]. The WGS assembly is the entire shotgun sequencing reads assembled into contigs including euchromatic and heterochromatic regions, even when not assembled into gapped contigs or not anchored on chromosomes yet. The regions enriched in TR are mostly not anchored, although TR and in particular satDNA are present in WGS due to their abundance in the genome.

**Figure 1:**
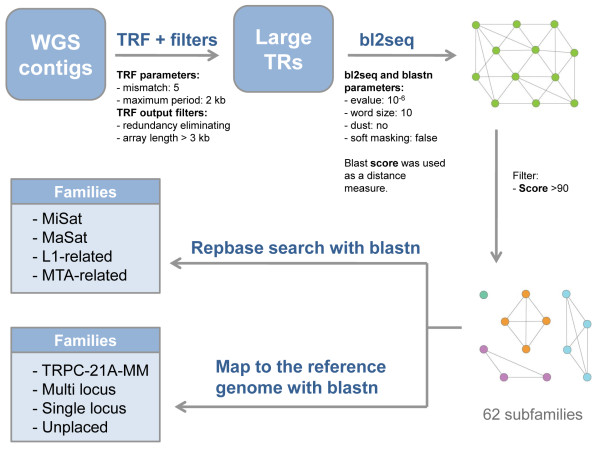
**Overview of the large tandem repeats analysis**. For each program only parameters that were changed are shown. The "blastn" was used for the "Repbase" search and for the genome mapping with parameters identical to "bl2seq". TR family names are given according to the Table 2. The complete description of the workflow is given in Results and Methods.

To identify all TR with unit size up to 2 kb we used TRF (Tandem Repeat Finder [23]). The initial raw TRF output contains data redundancy due to nested repeats and repeats with the same coordinates but different unit sizes. To eliminate this redundancy all nested repeats with array length less than the parent array were removed. In case of same coordinates an array with a longer unit size was removed. Both in MGSC (~3%) and Celera (~5%) WGS the amount of non-redundant TR is less than the experimentally determined amount of the MaSat alone (~8%) [24], indicating that even in WGS data sets TR remain underrepresented (Table 1). Since the mouse genome is enriched with micro- and minisatellites [21], we tried to get rid of them with a filter that excluded any array less than 3 kb. In both WGS collections we found 941 large TR (Table 1), which were further grouped into families due to sequence similarity (Figure 1).

**Table 1:** Tandem repeats in mouse WGS assemblies

Assembly	GPID	Size (bp)	Contigs	TR (all)	% of assembly	TR (> 3 kb)
MGSC WGS	13183	2,477,633,597	224,713	849,466	2.9%	157
Celera WGS	11785	3,003,109,157	837,963	1,084,552	5.0%	784
Total WGS		5,480,742,754	1,062,676	1,934,018	3.8%	941

GPID - NCBI Genome Project ID. TR (all) - total amount found in assembly; MGSC - the mouse genome sequencing consortium.

### Families and superfamilies

Each pair of arrays was compared by bl2seq program, and the score value was used as a measure of TR sequence similarity. Two tandem repeats were placed in the same subfamily if they had a bl2seq match with score greater than 90. This subdivided the TR into 62 subfamilies. We used the Blast search versus rodent Repbase repeat collection to check the similarity with known mouse repeats. This search determined only two known mouse satDNA: 715 arrays (~76%) represent pericentromeric MaSat and 21 (~2%) represent centromeric MiSat family (Table 2). The rest of the TR families are not present in Repbase; therefore, they were named according to their structure and genome position. For two families (C4, C5 in Table 2) the published nomenclature was used: single locus (SL) family for arrays found only once in the reference genome, whereas multi locus (ML) family for arrays found at more than one locus [5]. A subfamily name includes the letters TR (Tandem Repeat), genomic position (if known), minimal unit size in bp, index letter if there is more than one TR with similar unit size (A, B, etc), and suffix MM (*Mus Musculus*), with the latter present only in the tables and figures.

**Table 2:** Mouse large tandem repeats classification

Superfamily	N	Family	Genome Position	Arrays	% of TR	Subfamilies
A. Centromeric	1	MiSat	Cen*	21	2.2	1
B. Pericentromeric	2	MaSat	periCen*	715	76.0	1
C. Heterogeneous	3	TRPC-21A-MM	periCen	50	5.3	1
	4	Multi locus	Any	57	6.0	20
	5	Single locus	Any	56	6.0	29
	6	Unplaced	Absent	11	1.2	8
D. TE-related	7	MTA related	Any	15	1.6	1
	8	L1_MM related	Any	16	1.7	1

Eight families of the TR found in WGS were combined in four superfamilies (A-D). Families are formed according to sequence similarity and/or position in the reference genome. Arrays - the number of TR arrays found in WGS; % of TR - percent of all TR found in WGS; Subfamilies - number of subfamilies in family. (*) - MaSat and MiSat position is determined by *in situ *data published; (D) - Tandem repeats related to transposable elements (TE); (MTA) - mouse transcript retrotransposon, (L1) - L1_MM.

The characteristic feature of superfamily "C" is the prominent variability of the TR, which could be divided into subfamilies. The most abundant is TRPC-21A, which has a strictly pericentromeric location (Table 2, C3). Multi and single locus families each represent ~6% of the TR dataset. Some of the TR arrays (~1%) from Unplaced family (UnP, Table 2, C6), which have a distinct monomer and relatively long arrays, are not found in the reference genome.

The superfamily "D" is formed by MTA-related and L1-related families (~3% together), which show structural characteristics related to dispersed transposable elements (TE), but are tandemly organized, and have several features quite distinct from the most members of the set (Table 2, D7, D8).

**Figure 2:**
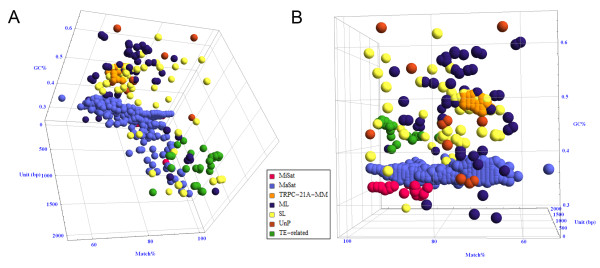
**TR arrays distribution graph**. The graph of tandem repeat arrays distribution was done in Mathematica™ 7.0. Each circle represents one array found in WGS assemblies. Each family was colored according to the Table 2: centromeric MiSat (magenta); pericentromeric MaSat (blue); TRPC-21A-MM (orange); heterogeneous multi locus (ML, indigo); heterogeneous single locus (SL, yellow); heterogeneous Unplaced (UnP, burnt orange); TE-related tandem repeats (TE, green). X axis - monomer length (bp) up to 2 kb; Y axis - GC-content is normalized to 1; Z axis - similarity between monomers. A and B - different projections of the same graph.

The relationship between families depending on monomer length, the degree of unit similarity and GC content are shown in the graph (Figure 2). The most clear and compact cloud is formed by MaSat arrays though it is not as uniform as might have been expected from the experimental studies [24,25]. MiSat cloud is in proximity to the MaSat but forms a distinct group. In the area of relatively short monomer unit, two defined clouds of TRPC-21A and other multi locus TR are visible. The transposon-related TR form a loose cloud in the region of long monomer units. Arrays from SL and UnP families are scattered throughout the whole plot. It is likely that additional data from oncoming mouse genome resequencing could improve the classification of SL and UnP families.

### Chromosome ends

Even for human, the best assembled mammalian genome, only chromosomes 8 and X have the higher-order repeat units known to be at the centromeric region [8,26,27]. The large regions of classical heterochromatin are poorly assembled [6], and for the mouse genome even less is known. Mouse telocentric chromosomes have extended TR arrays at the ends. That is the reason why these regions are difficult to assemble and chromosomes end abruptly in 3 Mb gaps reserved for centromeric regions.

We identified what kinds of TR are preceding these gaps (Table 3). The ends assembly does not allow to find TR on all chromosomes, so we determine the distance from the gap to the first gene (Additional file 1, Table S1). Only two assemblies end up in MaSat arrays: chromosomes 9 and 11. Four assemblies end up in the newly found TRPC-21A (chromosomes 3, 4, 16 and 17). On chromosomes 4 and 17 the arrays of TR-22A and TR-27A are followed by TRPC-21A. TR-22A arrays are also found at the very ends of chromosomes 6 and 18. We found out that only eight chromosome ends contain TR arrays and six of them are distinct from the pericentromeric MaSat.

**Table 3:** TR arrays in the region adjusted to centromeric gap

Chromosome	TR subfamily	Array length (kb)	Coordinates (bp)
3	TRPC-21A-MM	33.6	3000001-3033629
4	TRPC-21A-MM	7.0	3006469-3013522
4	TR-22A-MM	4.9	3104899-3109811
6	TR-22A-MM	9.9	3082006-3091879
9	MaSat	38.4	3000003-3038419
11	MaSat	3.9	3000004-3003872
16	TRPC-21A-MM	9.0	3232335-3241336
17	TRPC-21A-MM	32.5	3006399-3038945
17	TR-27A-MM	4.6	3070530-3075093
18	TR-22A-MM	8.0	3112790-3120776

Only TR with the array more than 3 kb in the distance up to 2 Mb from the centromeric gap is shown. TR - TR name is given according to Tables 4 and 5. Coordinates - the array position on chromosome.

### MiSat (minor satellite) and MaSat (major satellite) families

The previous experimental data indicated the sequence uniformity of mouse satDNA, i.e. MaSat monomers variability is less than 5% [25], and ~5.6% variation is found between MiSat monomers [28]. MaSat and MiSat are both AT-rich (64% and 66% respectively), and share stretches of sequences with 83% homology [16]. MiSat arrays were not found in the assembled reference genome. However, Chromosome Unknown (ChrUn) contains MiSat (Additional file 1, Table S2). Centromeric position of MiSat in Table 2 is given according to fluorescent *in situ *hybridisation (FISH) [29-31]. All the MiSat arrays (the longest array is ~6 kb) are AT-rich, with GC content no more than 33%. Monomer variability of MiSat family is the lowest of all families except TE-related superfamily. In accordance with the data published [18,20,28,32] and low monomer variability MiSat arrays do not have a prominent HOR structure. One third of the arrays have the 120 bp monomer unit reported for MiSat [14,28,32]. The rest has units of 112 bp, 223 bp, 232 bp and one of the units is 1054 bp. The unit difference may be a base for the HOR structure, but the limited number of MiSat arrays found in WGS makes it difficult to draw conclusions on this point right now.

**Figure 3:**
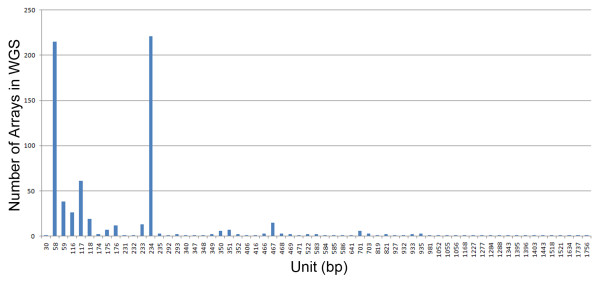
**MaSat unit length distribution**. X axis - unit length (bp); Y axis - number of the arrays with correspondent unit. The detailed data are shown in Additional file 2. Two main peaks represent 58-59 bp and 234 bp units; presence of larger units can be interpreted as the HOR structure for MaSat.

The pericentromeric AT-rich MaSat is formed by 234 bp heterotetramer that consists of four different 58-60 bp monomers with common motif [24]. MaSat is the most abundant family in WGS (Table 2). Very few MaSat arrays found in the WGS exceed 10 kb, with the longest being ~23 kb (Additional file 2, NN 234 and 316). The array of 38 kb is found at the end of chromosome 9 in the reference genome (Table 3). This feature, the array length, differs from the human genome, where alpha satDNA are assembled in arrays with length > 100 kb [6]. The MaSat family has GC content no more than 37% and the mean monomer variability of 30%. The MaSat has two common unit size variants: 35% of arrays have the experimentally described 58-59 bp monomer [24] and 31% have the 234 bp classical monomer (Figure 3).

MaSat arrays with short monomers have the most prominent variability (~30% for 58 bp unit). Arrays with 234 bp monomer show the lowest rate of the variability, with a mean of ~15% (NN 397-617 in Additional file 2). Very few of the arrays have variability about 5%. Thus, bioinformatics approach does not confirm the high degree of MaSat sequence conservation that was concluded from the experimental data [25].

**Figure 4:**
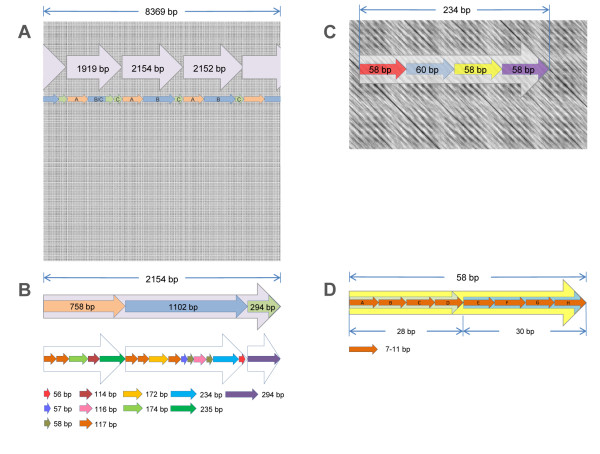
**MaSat HOR structure**. A: The dot plot of the MaSat array N707 (Additional file 2); a window size is 13 bp, sequence similarity is shown in gray scale. HOR units are shown as arrows with indicated length; smaller arrows indicate HOR subunits; different colors and letters indicate subunits variants. B: 2154 bp HOR unit structure; the color code for different units is shown. C: The structure of conventional MaSat 234 bp heterotetramer. D: 58 bp unit is built of 28 bp and 30 bp subunits consisting of 7-11 bp subunits; letters indicate subunits variants.

The high rate of the unit variability suggests the existence of a HOR structure in the array. This was checked with a dot-plot similarity analysis where the sequence is self-compared with the fixed 13 bp window (Figure 4). A degree of similarity is indicated by a greyscale where a darker grey represents higher degree of similarity. Therefore, repeated units with high similarity look like diagonal lines, and repeated motifs look like square patterns. We found that about 60% of MaSat arrays have a HOR structure with a clear "tartan" pattern (Figure 4A). A conservative 234 bp heterotetramer (58+60+58+58 bp units) is visible at higher magnification (Figure 4C). Moreover, each unit consists of two less conservative 28 bp and 30 bp subunits (Figure 4D).

TRF output contained MaSat arrays with a unit size of more than 1000 bp (Figure 3; Additional file 2, NN 698-715). It is likely that MaSat has units even larger than 2 kb, which are not detected by the TRF search that was restricted to a maximal unit size of 2 kb. Nevertheless the black and white dot-plot with 51 bp window size demonstrates the overall difference between HORs in different MaSat arrays and confirms the existence of ~2 kb HOR (Additional file 3, Figure S1A, B).

A prominent difference between MaSat arrays could be expected from dot-plot analysis (Figure 4A). The form of MaSat cloud on Figure 2 also suggests that MaSat is not as uniform as it was previously thought [30]. We suppose that being cloned and assembled each MaSat array might come to the different chromosomes, and then chromosome specificity could be suspected for MaSat previously counted as uniform.

### TRPC-21A-MM family

The second largest family in WGS is TRPC-21A (Heterogeneous TR, family C3, Table 2). It is more GC-rich in comparison to MiSat and MaSat, but its monomer variability is nearly the same (Table 4). In four cases, when it was found in the assembled genome, it is localized to the very end of centromeric gap (Table 3). Only on chromosome 7 it is placed in the internal band (7D1, Table 4). Moreover, TRPC-21A arrays are found in ChrUn which contains mostly pericentromeric regions (indicated by PC suffix in TRPC-21A name).

**Table 4:** TRPC-21A-MM family

N	Unit (bp)	Chromo Bands	Arrays	GC%	Length (bp)	Var%
1	42*	3A2	1	49.3	4739	27
2	21	3A2, 4A2	7	50.1	5288	29
3	63	3A2,17A2	2	48.6	4417	29
4	42	16A2,17A2	2	48.2	29884	31
5	21	7D1,16A2,17A2	6	50.1	4956	29
6	21	3A2,4A2,17A2	7	48.9	7698	30
7	21	3A2,16A2,17A2	5	48.7	17198	31
8	21	3A2,7D1,16A2,17A2	18	49.5	15684	29
9	209	3A2,4A2,16A2,17A2	1	48.4	7481	29
10	21	3A2,4A2,7D1,16A2,17A2	1	49.4	8021	29

Arrays of the TRPC-21A-MM family (Heterogeneous superfamily, Table 2, C3). N - row index; Unit (bp) - minimal unit length; Chromo Bands - chromosomal positions in the reference genome; Arrays - number of arrays in WGS with similarity to those chromo bands; GC% - mean array GC content; Length (bp) - maximal array length; Var% - mean variability between monomers in array. All arrays have HOR and present in ChrUn, so correspondent columns are omitted from this table.

* this sequence was used for the FISH probes design.

All TRPC-21A arrays were divided into ten groups according to the similarity to the specific locus in the reference genome (Table 4). The longest array of ~30 kb (N35 in Additional file 1, Table S3) probably belongs to chromosome 17 due to the high sequence and length similarity with the array at the end of this chromosome (Table 3). Most arrays show similarity with the band 3A that has the large TRPC-21A field at the end of chromosome (Tables 3 and 4).

**Figure 5:**
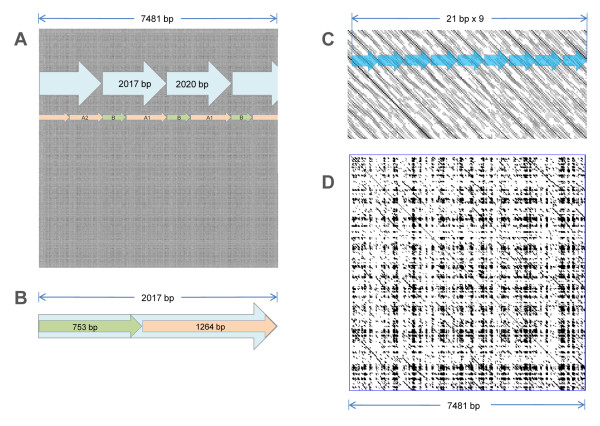
**TRPC-21A-MM HOR structure**. A: The similarity dot plot of TRPC-21A-MM array N50 (in Additional file 1, Table S3); a window size is 13 bp, sequence similarity is shown in gray scale. Units are shown as large arrows with length indicated. Smaller arrows indicate HOR subunits; colors indicate subunits variants. B; The structure of 2017 bp units, two subunits with corresponding size are indicated. C: 21 bp is the basic unit for TRPC-21A-MM as it is visible on dot plot at high magnification. Number of 21 monomers is indicated. Blue arrow represents one unit. D: The black and white dot plot with a window size of 51 bp and minimum 80% identity.

Arrays of TRPC-21A are organized by multiplication of the basic 21 bp unit, although TRPC-21A arrays are more homogeneous than MaSat arrays (Figure 5). All TRPC-21A arrays have a HOR structure on dot-plot. In this case even 60-mer units appeared (Figure 5A). PCR with specific primers on the template of total *M. musculus *DNA gave the ladder for TRPC-21A as well as for MaSat, indicating the characteristic feature of the satDNA, also caused by variable monomers organized in HOR (data not shown).

All the features of TRPC-21A are those of a "big classical" satDNA such as human satellites 1-4 [33]. They are known to be chromosome-specific. For example, the bulk of human satellite 3 (HS3) is located on chromosome 1, but it could be distinguished from HS3 on chromosome 9 [34]. To design a FISH probe for TRPC-21A we selected the array with a high similarity to the band 3A2.

### Multi locus, single locus and unplaced families

The Heterogeneous TR superfamily (Table 2) is classified into families according to their presence (ML, SL) or absence (UnP) in the reference genome (Tables 5, 6, and 7). The most abundant ML subfamily, TR-22A, was found in four loci in the reference genome; three are associated with centromeric gap (Table 3and 4A2, 6A2, 18B2 in Table 5) and one is located more distant from the centromeric gap (7A2, Table 5).

**Table 5:** Multi locus family

N	Subfamily	Unit (bp)	Chromo Bands	Arrays	GC%	Length	Var%	ChrUn	HOR
1	TR-22A-MM	22	4A2, 6A2, 7A2,18B2	9	58.1	12896	26	3	+
2	TR-4A-MM	4	1H6, 9F1, 9F3, XA4, XC2, XF5, YA2	6	33.1	7704	30	3	+
3	TR-27A-MM	27	14B,17A2	4	39.6	7073	27	0	+
4	TR-31C-MM	31	9A2*	4	49.5	13059	24	0	+
5	TR-18A-MM	18	14A2, XF5	3	55.5	5644	27	1	+
6	TR-19B-MM	19	5G1.2,12D1	3	47.8	5852	19	0	+
7	TR-38C-MM	38	12B1,13A3.2	3	48.7	6797	26	0	+
8	TR-57A-MM	57	5C2,7F5, 8A1.2, 10D3, 12F1, 14B, 16C2, 17A2, XA6	3	52.3	5619	21	0	+
9	TR-4B-MM	4	1H6, XA4, XC2	2	46.2	4161	36	3	+
10	TR-6A-MM	6	5C2, XC2, XF3, XF5	2	60.2	6649	39	0	+
11	TR-16A-MM	16	6E2,8A1.2, 16C3.2	2	54.2	6765	17	0	+
12	TR-20A-MM	20	XA4, XF5	2	45.5	3604	31	0	+
13	TR-31A-MM	31	7D1,8C1	2	50.6	6558	20	3	+
14	TR-31B-MM	31	7D1,14A2	2	53.2	4922	19	1	+
15	TR-58A-MM	58	6B2.2,6C2, 6F3, 13B1	2	50.5	4658	34	0	+
16	TR-1521A-MM	1521	7F3, XA1.2	2	44.6	3213	11	1	+
17	TR-30A-MM	30	5B3, 17B1	1	46.3	3912	37	0	+
18	TR-81A-MM	81	14B,17A2	1	40.0	3483	31	0	+
19	TR-1164A-MM	1164	XC2, XD	1	48.6	3333	19	0	-
20	TR-1595A-MM	1595	XA3.2, XA6	1	38.8	3007	16	0	-
21	TR-1149A-MM	1149	XF1, XF3	1	45.8	5463	13	5	-
22	TR-1527A-MM	1527	XC2, XD	1	46.0	3120	12	0	+

Subfamilies ordered by number of arrays in WGS and then by unit length. N - row index; Unit (bp) - minimal unit length; Chromo Bands - chromosomal positions in the reference genome; Arrays - number of arrays of each subfamily found in both WGS; GC% - mean array GC content; Length (bp) - max array length; Var% - mean variability between monomers in array. ChrUn - number of arrays found in ChrUn; HOR - presence of HOR.

* There are several distinct arrays of TR-31C-MM in 9A2 locus in the reference genome.

**Table 6:** Single locus family

N	Subfamily	Unit (bp)	Chromo Bands	Arrays	GC%	Length	Var%	ChrUn	HOR
1	TR-17A-MM	17	17D	5	43.2	10312	33	0	+
2	TR-54B-MM	54	XA1.2	5	47.9	11978	31	0	-
3	TR-29A-MM	29	2F1	4	50.3	4175	26	0	+
4	TR-734A-MM	734	XC2	3	37.0	9507	22	0	+
5	TR-1870A-MM	1870	7D1	3	44.6	5795	3	8	-
6	TR-19A-MM	19	18A2	2	49.7	4883	32	0	+
7	TR-34A-MM	34	12F2	2	55.2	3354	7	0	-
8	TR-38A-MM	38	8C1	2	42.7	5426	25	0	+
9	TR-38B-MM	38	8C1	2	42.0	6113	25	0	+
10	TR-54A-MM	54	XA1.2	2	48.1	10744	30	0	-
11	TR-100A-MM	100	XA1.2	2	44.0	4364	26	0	-
12	TR-234A-MM	234	3F2.2	2	59.4	6878	5	0	-
13	TR-23A-MM	23	17B1	1	43.4	6018	13	0	-
14	TR-24C-MM	24	12F1	1	52.5	3298	9	0	-
15	TR-29B-MM	29	2F1	1	50.0	15896	30	0	+
16	TR-31D-MM	31	12A1.2	1	55.9	5175	24	1	+
17	TR-33A-MM	33	7B1	1	53.4	3601	17	0	-
18	TR-39A-MM	39	1C1.2	1	39.2	3387	5	0	-
19	TR-40A-MM	40	15A2	1	62.6	6612	21	0	+
20	TR-44A-MM	44	2H3	1	36.3	3016	9	0	-
21	TR-48A-MM	48	14D3	1	50.7	6603	28	0	-
22	TR-56A-MM	56	XA1.2	1	42.9	4194	22	0	+
23	TR-84A-MM	84	7E2	1	48.5	3040	9	0	-
24	TR-93A-MM	93	XC2	1	51.3	3124	24	3	-
25	TR-111A-MM	111	9F4	1	52.4	3347	22	0	-
26	TR-168A-MM	168	XA1.2	1	41.9	4046	23	0	-
27	TR-297A-MM	297	17E1.2	1	56.2	3100	19	0	-
28	TR-321A-MM	321	11E2	1	42.3	3152	21	0	+
29	TR-814A-MM	814	5A2	1	45.5	3175	3	0	-
30	TR-1146A-MM	1146	19B	1	47.5	3056	14	0	-
31	TR-1284A-MM	1284	10C3	1	42.3	5239	14	0	-
32	TR-1384A-MM	1384	XF1	1	25.8	3665	8	0	-
33	TR-1872A-MM	1872	9A4	1	42.5	4285	17	0	-
34	TR-1908A-MM	1908	2A2	1	39.8	4126	14	0	+

Subfamilies ordered by number of arrays in WGS and then by unit length. N - row index; Unit (bp) - minimal unit length; Chromo Bands - chromosomal positions in the reference genome; Arrays - number of arrays of each subfamily found in both WGS; GC% - mean array GC content; Length (bp) - max array length; Var% - mean variability between monomers in array. ChrUn - number of arrays found in ChrUn; HOR - presence of HOR.

**Table 7:** Unplaced family

N	Subfamily	Unit (bp)	Arrays	GC%	Length (bp)	Var%	HOR	ChrUn	WGS Chr
1	TR-24B-MM	24	3	34.4	7636	28	+	2	11
2	TR-24A-MM	24	2	45.6	3540	22	+	2	Un
3	TR-13A-MM	13	1	56.0	4477	15	+	0	6
4	TR-27B-MM	27	1	63.5	3452	36	+	1	Un
5	TR-28A-MM	28	1	41.2	3195	23	+	1	Un
6	TR-36A-MM	36	1	64.0	3003	8	+	0	19
7	TR-102A-MM	102	1	45.7	3698	31	-	0	X
8	TR-624A-MM	624	1	43.3	3297	2	-	0	Un

Subfamilies ordered by number of arrays in WGS and then by unit length. N - row index; Unit (bp) - minimal unit length; Arrays - number of arrays in WGS; GC% - mean array GC%; Length (bp) - max array length; Var% - mean variability between monomers in array. HOR - presence of HOR; ChrUn - arrays found in ChrUn; WGS Chr - chromosome source from WGS contig description.

ML TR-4A consists of a very short AT-rich unit. About a half of the ML subfamilies is present on the sex chromosomes (Table 5). It could be explained by more accurate assembly of the heterochromatic regions on the sex chromosomes relative to autosomes. On the other hand, it is known that the sex chromosomes have unique DNA repeats [35-37] and ML TR-4A can be one of them.

Despite the minimal sequence similarity, several ML and SL subfamilies have similar GC-content, unit size, and array variability, forming three visually distinct groups (clouds) on the graph: GC-rich, AT-rich, and GC-neutral (Figure 2).

TR-22A subfamily is the core of GC-rich cloud in the area of 55-60% GC, while TR-6A, TR-57A, TR-16A and TR-31B are closely adjoined. At least one subfamily from SL, TR-31D, also belongs to this group (Additional file 1, Table S3).

The core of AT-rich cloud in the area of 40-45% GC is formed by subfamilies from SL (TR-17A, TR-38A, TR-39A and other). However, several ML, such as TR-81A, also gravitate towards this cloud. Several UnP arrays (TR-24B, TR-28A and other) belong to this group as well (Figure 2 Table 7). Two of subfamilies from AT-rich cloud (TR-39A, TR-44A) are embedded into the MaSat cloud or gravitate to MaSat (TR-81A, TR-4A).

Several ML subfamilies (TR-31A, TR-58A) and SL subfamilies (TR-54B, TR-29A, TR-24C) arrays gravitate upon TRPC-21A and form cloud with neutral GC-content (45-55% GC). Most of them have HOR and are present in ChrUn (Tables 5 and 6).

A number of ML arrays (Table 5, NN 19-22) and SL arrays (Table 6, NN 29-34) have a very long monomer of > 1 kb, although the structure of the long SL and ML TR does not show extensive similarity with known TEs. However, TR with long units that are classified as ML or SL families could be built on the base of very divergent or unknown TE. The existence of such TEs was predicted in vertebrate genomes [38].

A list of array positions in WGS for the Heterogeneous superfamily is given in the Additional file 1, Table S3.

### Transposable elements related tandem repeats

Two families have structural similarity to transposable elements (TE, superfamily D, Table 2). The arrays are formed by the large monomers with a low degree of diversity and similar GC-content in both families (Additional file 1, Table S4).

**Figure 6:**
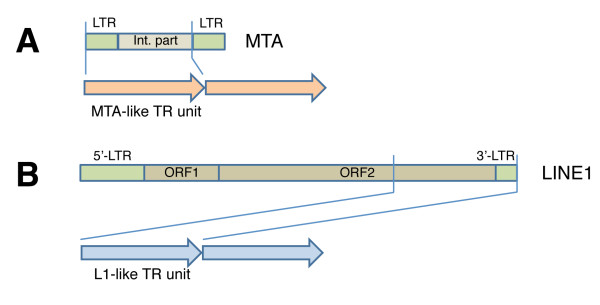
**Structure of TE-related tandem repeats**. A: The general scheme of MTA related TR family; regions of MTA are denoted, and a fragment of TR unit is marked. B: The general scheme of L1 related TR family; L1 regions are denoted, and a fragment of TR unit is marked.

First family, TR-MTA, is formed by MTA fragments: MTa, MaLR-LTR, Mammalian apparent LTR-retrotransposons in Repbase (Figure 6A). Second family, L1-related family is formed by part of the ORF2 and 3'LTR (Figure 6B).

MTA transposons have structural similarities to endogenous retroviruses, namely ERV3, and are related to THE1 in humans [39]. Endogenous retroviruses by themselves comprise ~10% of the mouse genome [40]. Over time, most MaLRs have diverged considerably from their consensus sequence, so their number is now estimated at 25-94,000 copies [39]. Preliminary analysis has not yet revealed significant similarities of the putative product of MTA ORF to any protein present in the databanks. The residual part of the ORF is now determined as internal part in MTA Repbase consensus and it is included in TR arrays.

**Figure 7:**
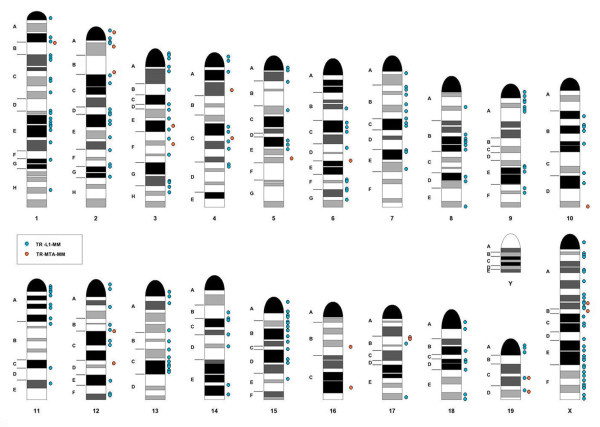
**Chromosomal location of TE-related tandem repeats**. Ideogram of mouse karyotype with MTA-like array positions indicated in orange, L1-like array positions indicated in blue. For ideogram description see the Methods section.

In order to map TE-related arrays to the reference genome two rules were applied. First, a TR hit at a chromosome locus counts as positive only when the alignment length is more than 2850 bp (95% from the original TR array limit of 3 kb). Second, a hit is counted as a single when the distance between two hits is less than 150 bp (5%). After applying these rules 284 hits with precise positions remained (Additional file 1, Table S5). Most of the loci found for TR-L1 family do not exceed 5 kb. For TR-MTA family we found two loci with array length about 10 kb. All loci were displayed on the banded chromosomes. There is no obvious regularity in TR-MTA family distribution, probably due to the limited amount of the arrays found (Figure 7, orange). The TR-L1 family is enriched in heterochromatic bands and the concentration on chromosome X is visible (Figure 7, blue). At the same time no TE-related TR are found on Y chromosome. Validation of these findings by FISH is technically challenging, because the LTRs of other retroelements may obscure the results.

### Tandem repeat position defined by FISH

**Figure 8:**
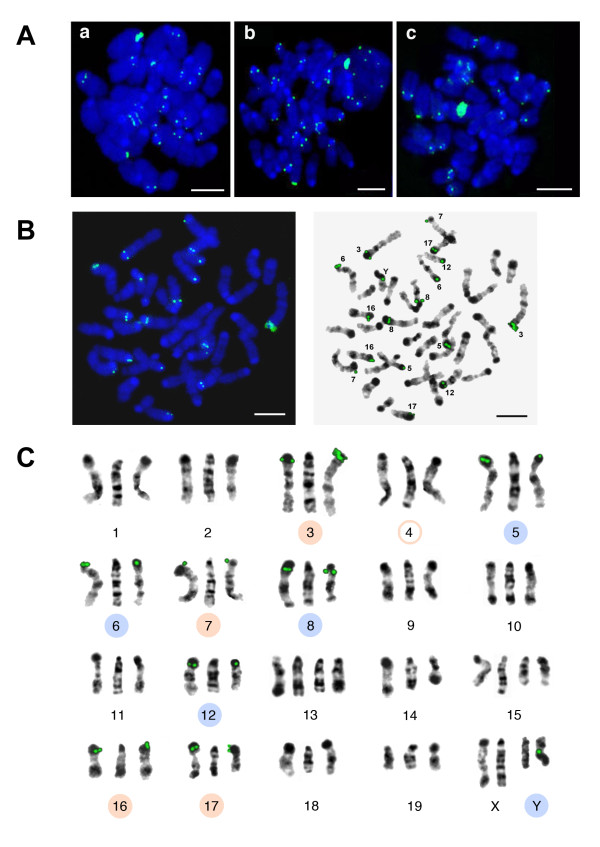
**Fluorescent *in situ *hybridization (FISH) with TRPC-21A-MM short probe**. A: bone marrow metaphase plates; B: one of the metaphase sets of chromosomes negative DAPI-banded, numbers of signal bearing chromosomes are indicated. For A and B: DAPI in blue, FISH signal in green; bar - 5 μm. C: all chromosomes karyotyped. In each group the middle image is G-banded mouse chromosome from atlas [41], the side negative DAPI-banded chromosomes are from the plate shown on B. Nine chromosomes with the label are indicated by circles; four chromosomes, with *in situ *signal that confirmed *in silico *prediction, are indicated by orange circle. The assembled chromosome 4 has short TRPC-21A-MM array but does not have signal (indicated by empty orange circle). Chromosome 7 has signal in pericentromeric region instead of predicted *in silico *7D1 band.

Bioinformatics predictions about the positions of newly-found TR were checked by *in situ *experiments. We did not expect to obtain *in situ *the full correspondence of TR positions found *in silico*, since the assembly of heterochromatic part of the reference genome is far from being complete. Nevertheless, *in silico *chromosome locations should be included in the set of the *in situ *labelled chromosomes. Monomer units from three classes were selected for probe design (see Methods section). All probe sequences with a short description shown in Additional file 1, Table S6. In the reference genome, TRPC-21A has predicted *in silico *pericentromeric location on four chromosomes (Table 3 and 4) and TRPC-21A arrays were found in ChrUn, which contains mostly pericentromeric regions; therefore, additional chromosomes have to be labelled in the same region. Single strand dimer labelled from both ends yielded signal on nine chromosomes: 3, 5, 6, 7, 8, 12, 16, 17, and Y. The largest signal belongs to chromosome 3 on all chromosome spreads. In each case the label was at the pericentromeric regions except the Y (Figure 8). Four chromosomes predicted as TRPC-21A bearing (Table 4) are in the set of *in situ *labelled chromosomes. Chromosome 4 has short TRPC-21A array *in silico *(Table 3) but it lacks any signal, probably due to the wrong assembly. Other discrepancy is the pericentromeric signal seen on chromosome 7, while *in silico *TRPC-21A mapped to the internal 7D1 band. Instead, Y chromosome has the internal signal, which could be explained by the unique repeats content of the sex chromosomes [20].

**Figure 9:**
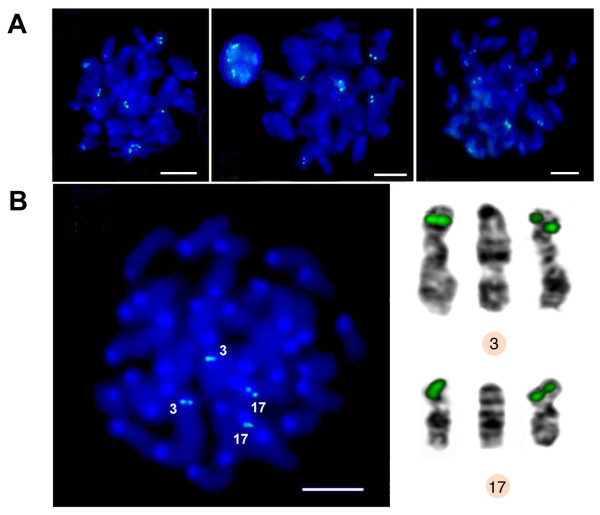
**FISH with TRPC-21A-MM long probe**. A: bone marrow metaphase plates; B: chromosome analysis on the metaphase plates; the numbers are indicated (left). DAPI in blue, FISH signal in green. *In situ *positive chromosomes (negative DAPI-banded) are shown (right); bar - 5 μm.

The HOR structure of TRPC-21A suggests chromosome-specific variants (Figure 5). The next probe was based on the array fragment from chromosome 3. The probe is a double stranded ~150 bp sequence with additional ~20 bp flanking sequences. Flanks give the possibility to label probe by PCR (Additional file 3, Figure S2). This probe has a strong signal on chromosomes 3 and 17 according to the position of large TRPC-21A arrays at the ends of these chromosomes in the reference genome (Table 3, Figure 9). We suppose that probes designed on the basis of TRPC-21A variants could be specific for other chromosomes.

**Figure 10:**
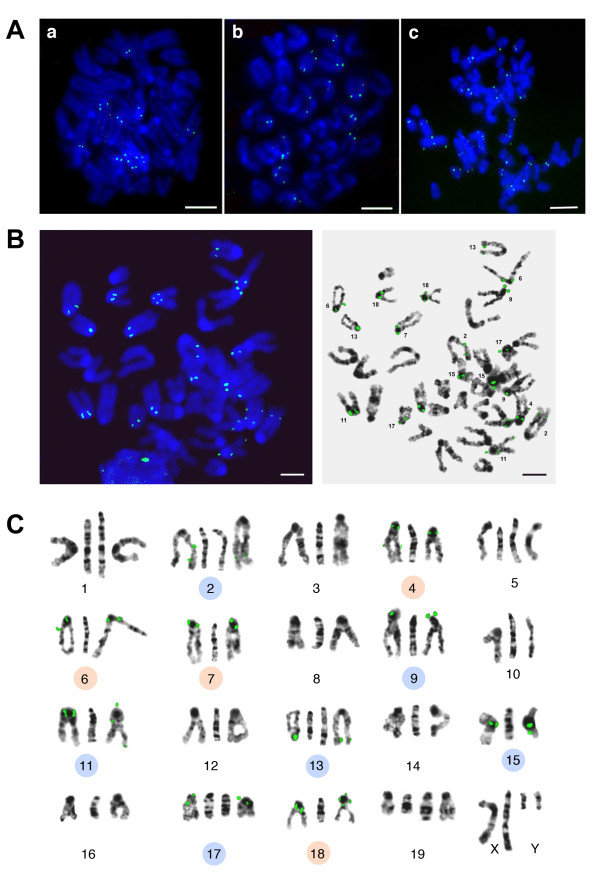
**FISH with TR-22A-MM probe**. A: Primary bone marrow metaphase plates (a, b) and metaphase plate from cell line L929 (c). DAPI is blue, FISH signal is green; bar - 5 μm. B: one of the bone marrow metaphase plates with chromosome numbers indicated. Bar - 5 μm. C: in each chromosome group the middle image is G-banded mouse chromosome from atlas [41], the side (left and right) negative DAPI-banded chromosomes are from the plate shown on B. Ten chromosomes with the FISH signal are indicated by circles, four chromosomes with *in situ *signal that confirmed *in silico *prediction are indicated by orange circles.

TR-22A (ML, C4 in Table 2) was chosen for the probe design due to its abundance in the reference genome as well as in ChrUn (Table 5). The monomeric single strand probe labelled from both ends is hybridized to ten chromosomes, four of them predicted as TR-22A bearing (Table 5). In this case the main part of the signal is located at the pericentromeric regions (chromosomes 2, 6, 7, 9, 11, 17, 18), with additional signals located on the arms of chromosomes 2 and 15, and in the subtelomeric region of chromosome 13. In each case signals are located in heterochromatic dark bands (Figure 10). The signal is stronger on L929 chromosome spreads comparing with the signal on normal bone marrow cells (Figure 10Ac). It could be explained by known chromosome polyploidy and rearrangements within heterochromatic regions in L929 cells [30,41]. There is no obvious main signal on any chromosome spread, so the design of chromosome-specific probe on the base of TR-22A could be more complicated and, moreover, the arrays at the ends of chromosomes 4, 6 and 18 in the reference genome do not exceed 10kb (Table 3).

**Figure 11:**
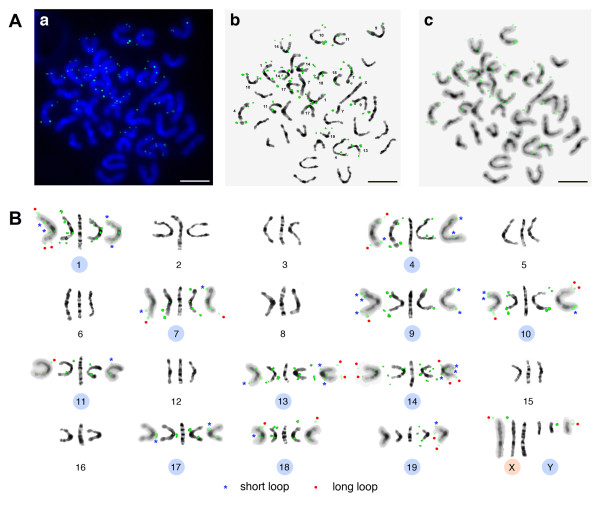
**High resolution FISH with TR-54B-MM probe**. A: (a) bone marrow prophase chromosome spread. DAPI in blue, FISH signal in green; bar - 5 μm; additionally shown a negative DAPI-banded central core of chromosomes (b) and "fuzzy" structure of whole DAPI-stained chromosomes (c). B: In each group the middle image is from atlas [41], the side (left and right) negative DAPI-banded chromosomes are from the plate. Ten chromosomes with the label are indicated by circles; chromosome X bearing the label in accordance with *in silico *prediction indicated by orange circle. The labels on the short chromatin loops marked with blue asterisks; label belongs to the long loops marked with red circles.

Finally, the SL TR-54B (C5, Table 2) was selected due to the abundance of its arrays at the XA1.2 pericentromeric band. A double strand dimer probe was designed and labelled by PCR. About half of all signals obtained in the late prophase chromosome spreads belong to the long loops emerged from subtelomeric regions of chromosomes during inevitable osmotic shock, which is a necessary step during chromosome-spread isolation [42,43]. The signal on the chromosome X is located at the predicted region. However, this signal as well as most of the rest could only be recognized on "fuzzy" chromosomes, when all the DAPI stained material is visible but bands are obscure. In contrast to the reference genome assembly, TR-54B is not a single locus TR, because about fifty signals in total are visible on chromosome spreads (Figure 11). The further mapping of TR-54B using additional probe for the subtelomeric region is required to clarify its exact location.

## Discussion

The computation approaches to the genome-wide TR analysis gradually appear with the genome sequencing advanced [5,6,44-46]. At the chromosomal level TR can be of profound structural as well as evolutionary importance, since genomic regions with a high density of TR, e.g., telomeric, centromeric, and heterochromatic regions, often have specific properties such as alternative DNA structure and packaging [47-49]. At the nuclear level of organization, constitutive heterochromatin may help maintain the proper spatial relationships necessary for the efficient operation of the cell through the stages of mitosis and meiosis. In the interphase nucleus satDNA have one property in common despite their species specificity, namely heterochromatization, which involves RNA interference-mediated chromatin modifications [2,3,50-54]. The strand-specific burst in transcription of pericentromeric satellites is required for chromocenters formation in early mouse development. Specific expression dynamics of MaSat repeats, together with their strand-specific control, represent necessary mechanisms during a critical time window in pre-implantation development that are of key importance to consolidate the maternal and to set up the paternal heterochromatic state at pericentromeric domains [55]. Such an important and crucial finding is based on the known sequence of the mouse MaSat. Most of the other mouse TR could not be tested in similar experiments being undescribed and unclassified.

### Mouse major satellite

The proportion of MaSat in a total mouse DNA preparation is about 8%, and it is higher than the amount of satDNA found in total DNA preparations from rat and human [24]. MaSat is located near chromosome centromeres [56]. The most wide-spread opinion based on experimental data is the high degree of MiSat and MaSat sequence conservation exists across the telocentric domain of all mouse chromosomes. The earlier publications do not confirm MaSat uniformity. There are data for both short range [57] and long-range periodicity in MaSat [58]. EcoRII digest breaks MaSat into fragments, which form a series of bands on gel electrophoresis (ladder). The DNA in the strongest band was 220-260 bp and the other bands were the multiples of this length. The stronger bands of the minor patterns fall half-way between the bands of the main pattern, and the smallest is 120 to 130 nucleotide pairs long [58]. Monomers of the correspondent length are the third in representation among MaSat monomers in the arrays (Figure 3). The sequence is shown to be based on a repeating unit less than 20 bp in length. Four major oligonucleotides were identified, all of which could derive from an original sequence d(GA_5_TGA) for the light strand [57]. Short units of the size similar to the reported oligonucleotides could be tracked by MaSat dot-plot analysis (Figure 4D). In contrast to proposed MaSat uniformity based on limited experimental data [25], our results indicate that its monomers variation is quite high. Despite the abundance of MaSat in TRF outputs, the majority of MaSat is unplaced and in all likelihood will be placed in 3 Mb centromeric gaps on each chromosome. We suppose that MaSat arrays could be chromosome-specific and thus may come to different chromosomes during attempts to fill centromeric gap. For this purpose the probes based on different MaSat variants could be designed and checked by FISH.

### Mouse minor satellite

There were previous attempts to find MiSat chromosome-specific variants. MiSat specificity has been shown to chromosome 2 with synthetic oligonucleotide probes and Southern hybridization [59]. Oligonucleotide probes that specifically detect sequence variations were found in some cloned MiSat fragments, and they detected a limited subset of MiSat arrays using pulse-gel electrophoresis with Southern hybridization and PRINS (primed *in situ *hybridization). Mostly prominent label corresponded to chromosomes 1 and 14 [28]. The existence of a chromosome specific MiSat implies that the rate of sequence exchanges between non-homologous chromosomes relative to the rate of exchange between homologous chromosomes is much lower than was postulated previously. Based on these results the suggestion was made that the high degree of sequence homogeneity of both known mouse satDNA may reflect recent common ancestry [28]. Still, none of these probes have been worked up to be a reliable cytogenetic marker. Since only a few MiSat arrays were found in WGS (Additional file 1, Table S2), this does not give much hope to a successful design of a chromosome-specific probe with purely bioinformatics approach.

### Classical satellites

Big classical satDNA are well known and were studied extensively. Human satDNA 1-4 (HS 1-4) are based on a "simple" 5-6 bp motif and HS3 is mostly well investigated [10,11]. HS3 was found in pericentromeric regions of all chromosomes but 2, 6, 8, 11, 12, 18, 19, and X [60,61]. Chromosome-specific subfamilies of HS3 have been determined [62,63] and those that belongs to chromosome 1 (HS3-1) and 9 (HS3-9) are two of them [61].

Mouse TRPC-21A resembles human "classical" or "simple sequence" satellites in most features. The dot-plot at a high magnification suggests that units of ~5-7 bp could be distinguished inside the basic 21 bp monomer (grey lines between black ones on Figure 5C), although the degree of diversity demands a special investigation to determine the exact oligonucleotide sequence. All of the TRPC-21A arrays contain HORs, it is common characteristic of classical satellites. Letters "PC" are included in the TRPC-21A name to indicate strictly pericentromeric location determined according to the relevant WGS position, position in the assembled genome (Tables 3 and 4) and confirmed by the FISH signal (Figures 8 and 9). The most prominent chromosome-specific probe was designed at the base of chromosome 3 variant of TRPC-21A (Table 4; Additional file 3, Figure S2; Additional file 1, Table S6).

TRPC-21A was the first tested, but among ML family some of the family members (TR-22A, TR-27A, and TR-31A) look promising for chromosomes-specific probes design due to HORs and their presence in the ChrUn. TR-22A and TR-27A were also found at the end of assembled chromosomes (Table 3), so the possibility to map them by FISH is quite high.

### GC content

It is notable that most of newly found subfamilies have GC content higher than MaSat and MiSat - the mean for TRPC-21A is ~50%, and even higher for ML family - ~57% (Figure 2). Both GC-rich and AT-rich satDNA are known in human and most of the high eukaryotes [64,65], hence our results cure the strange asymmetric satDNA distribution reported for mouse until now. The isochors (regions differ in GC content) have the functional significance for optimization of epigenetic genome regulation and supports the notion that noncoding DNA is important for orderly chromatin condensation and chromatin-mediated suppression of tissue-specific genes [66]. The absolute values of thermostability, bendability and ability to B ± Z transition correlated positively with the GC content, whereas curvature correlated negatively [67]. Although these conclusions were made on the base of introns and intergenic spacers as examples of noncoding DNA, TR of different GC and AT content may add to the isochoric genome structure due to its abundance in some regions.

### Bar code

Alpha-satDNA is the only functional DNA sequence associated with all naturally occurring human centromeres. Two distinct forms of alpha-satellite are recognized based on their organization and sequence properties. A large fraction of alpha-satellite is arranged into HOR arrays where corresponding monomers are organized as multimeric repeat units ranging in size from 3 to 5 Mb [68,69]. Human chromosome-specific probes based on alpha-satDNA [70,71], "classical" satDNA [34], and megasatellites [6] exist and are used in cytogenetic analysis. It appears that using human WGS and assembled genome a set of TR characteristic for each human chromosome could be found, suggesting that TR might provide a kind of "bar code" for each chromosome.

The lack of mouse chromosome-specific probes causes problems for most genome-connected studies, including studies in developmental biology. Using WGS we have identified 62 subfamily of large tandemly repeated DNA. The next step is to map most of them to check whether there is the chromosome specificity in the hybridization pattern. Probably, it will be possible to create individual chromosome "bar-code" set of probes to be used in cytogenetic analysis. We suggest that this "bar-code" describes the heterochromatin signature for each chromosome and these signatures help to arrange chromosomes in the nucleus in the specific order during development. Potentially, this "bar-code" or signature represents the hypothetical Master Development Program, previously attributed to the heterochromatic regions [72].

## Conclusions

Eight families including 62 subfamilies are found and characterized here by bioinformatics analysis. Most of them are more GC-rich than well known MaSat and MiSat. HOR structure was determined for some of them suggesting the existence of TR chromosome-specific variants. Probes for the representatives of three TR families were designed on the base of TR monomers units. *In situ *hybridization signal positions are in accordance with *in silico *predictions on the reference genome, although other chromosomes are labelled due to the poor assembly of the heterochromatic genome regions. A long probe based on chromosome 3 variant of TRPC-21A recognizes the longest fields of TR at the ends of chromosomes 3 and 17. No reliable cytogenetic probe was designed up to now. We suppose that with the future investigation of the newly characterized TR families it will be possible to determine the set of mouse chromosome-specific TR.

## Methods

### Sequence databases

Mouse sequences were obtained from NCBI ftp site in FASTA format: two WGS assemblies for projects AAHY and CAAA [73]; the reference genome assembly build 37.1 and Celera genome assembly build 37.1 [74]; MGSC genome assembly release 3 [75]. The genome banding annotation was obtained from the NCBI ftp site [76]. The Repbase database version 15.07 in FASTA format was obtained from [77]. To compile local blast databases we used blastdb program from BLAST+ suite with default parameters.

### Programs and search parameters used

Sequence alignments were performed using blastn and bl2seq from BLAST+ suite [78]. Several search parameters were changed to work with repetitive DNA: *max_target_seqs *(the maximum number of database sequences for which any alignment will be reported) and *num_descriptions *(the maximum number of one-line descriptions of significant database sequenced reported) were set to 10,000, *evalue *(expectation value threshold for saving hit) was set to 10^-16^, *word_size *for word finder algorithm was set to 10, *dust *(arguments to DUST filtering algorithm) was set to "no", *soft_masking *parameter (simple repeat filter) was set to "false". All other search parameters were set to default values. Tandem repeat search was performed using TRF [23]. Search parameter *mismatch *was set to 5; *maximum period size *was set to 2000. Other search parameters were set to the default values. Self dot-plot matrix computations were done with in-house software with two sets of parameters: (1) window size set to 13 bp and similarity indicated by gray-scale color from black (100% window match) to white (100% window mismatch); (2) window size set to 51 bp and similarity indicated by two colors: black for > 90% (MaSat arrays) or > 80% (TRPC-21A arrays) window match; white for corresponding mismatch. To store the mouse large tandem repeat collection we used MySQL 5.1 database. TRF output analysis was performed with custom Python scripts. 3D-plots were rendered with Mathematica™ 7.0. A coordinate representation of mouse chromosome ideogram [76] and band position of DNA repeats were used for chromosome ideogram drawing with custom Python script.

### TR analysis

To eliminate any redundant entries from the TRF output, all embedded TR arrays were discarded; in the case when two arrays had the same sequence coordinates a TR with a larger unit size was discarded. Overlapping arrays were considered as independent arrays. Repbase version 15.07 was used to compare TR with known repeats [79]. To remove false positive matches from Blast versus Repbase results, all matches that covered by repeats from Repbase less than 80% were discarded. Each pair of arrays was compared using bl2seq. We got a number of false-positive alignments due to the tandem nature of compared sequences. To remove false-positive or suspicious alignments we discarded all pair matches with a score less than 90. The remaining arrays were separated in subfamilies by Blast defined similarity. Two tandem repeats were placed in the same subfamily if they have a bl2seq match with score greater than 90. Finally, each subfamily checked by hand for errors. In several cases subfamily exact borders are fuzzy (TR-29A/B; TR-4A/B; TR-81A and TR-27A; TR-54A/B; TR-38A/B). Those subfamilies pairs can be joined in one bigger subfamily with less strict Blast parameters.

### Mouse genome databases comparison

We used three mouse genome assembles: the reference genome, the alternate (Celera) genome и MGSC genome assembly. Each genome has Chromosome Unknown (CrhUn) that contains all unplaced or unmapped contigs remained after assembly. TRF search with the parameters same as described has been applied to these 6 databases (Additional file 1, Table S7). There are prominent differences in total and large TR amount in different databases. It can be explained with a difference in the methodology of genome sequencing and assembly [80,81]. In the genome assembly process the additional sequence sources (e.g. clone based sequence) were used [21], which caused the difference in TR number found in WGS and genome assemblies. We used the reference genome build 37.1 as the most comprehensible, widely used and containing the largest amount of large TR to map newly found TR. The reference genome build 37.1 was assembled without WGS sequences and it has small ChrUn. Alternate (Celera) ChrUn was used to check the amount of TR found in WGS (Additional file 1, Table S7). We did not use MGSC genome and ChrUn assemblies as outdated with the lack of Y chromosome [21].

### Probe design

The probes 1 and 4 tested in FISH the probe were designed as follows. Fragment composed of several monomers with a total length ~150 bp was chosen from the most variable region of tandem array, and it was flanked by two different adapters (Additional file 1, Table S6 and Additional file 3, Figure S2). The probe was amplified with primers to adapters and labelled with biotine-dUTP by PCR: 95°C 15 sec, 60°C 30 sec, 72°C 30 sec, 20 circles. Probes 2 and 3 were synthesized as 3'-/5'-biotine labelled (Beagle, St. Petersburg, Russia).

### Mitotic chromosomes

Chromosome spreads from bone marrow cells were made according to the previously published method [82]. Colchicine (0.4 ml 0.04% solution) was injected intraperitoneally for 90 min before mice were sacrificed by cervical dislocation under anaesthesia. Bone marrow was washed out from legs tubular cylindrical bones with 75 mM KCl. Suspension was incubated 15 min in 37°C and centrifuged 5 min 1000 rpm. Pellet was fixed 15 min in cold fixative (methanol: acetic acid - 3:1) at 4°C. Suspension and centrifugation cycles were repeated three times. At last, the suspension was dropped on wet cold slides, which were air dried to get rid of the fixative. Metaphase plates of good quality were selected under microscope.

L929 cells were cultured in Petri dishes in full medium (DMEM+10% FCS) until 40-50% confluency. The cells were treated with colcemid (0.5 μg/ml) for 2 h before harvesting. After treatment with hypotonic solution (50 mM KCl: 1% Na citrate, 1: 1) for 20 min at 37°C the cells were fixed with acetic acid: methanol (1: 3), dropped onto ice-cold glass slides and air-dried. The slides were kept at -20°C until FISH was performed.

### FISH with double stranded probes

FISH with double stranded probes was done in the usual way [29]. The labeled probes were dissolved in the hybridization mixture (50 μg/μl sheared yeast total DNA, 50% formamid, 10% dextran sulphate, 2x SSC), loaded on a slide with cells, covered with a smaller cover slip, and sealed with rubber cement. Slides were denatured simultaneously on a hot-block at 75°C for 2 min. Hybridization was performed overnight at 37°C in a humid chamber. Post-hybridization washes were done at 42°C in 50% formamid for 10 min, twice in 2x SSC for 5 min, 0.5x SSC for 10 min and finally in 2xSSC for 10 min at room temperature. The slides were counterstained with 0.5 μg/ml DAPI in 2xSSC solution for 5 min, rinsed in 2xSSC, and mounted in Citifluor antifade solution (Citifluor Ltd, UK).

### FISH with single stranded oligonucleotide probes

FISH with single stranded probes was done according to a published protocol [83] with the following modifications. Oligonucleotides were synthesized as 3'-/5'-biotine labeled for probes 2 and 3 (Additional file 1, Table S6). After RNase and pepsin pretreatment, metaphase chromosome spreads were dehydrated in ethanol series and air-dried. Chromosomes were denatured for 2 min in 70% formamide, 2 ȕ SSC, at 65°C. After being dehydrated in an ice-cold ethanol series of washes, hybridization was performed for 12-16 h at 37°C. The hybridization solution contained 5 ng/ml probe, sheared yeast total DNA (50 μg/μl), 25% formamide, 4 × SSC. After hybridization, the slides were washed three times for 5 min in 2 × SSC at room temperature. For detection, preparations were incubated with fluorescein avidin D (Vector Laboratories) (5 μg/ml in 2 × SSC containing 5%BSA) for 40 min at room temperature. Then they were washed three times for 8 min in 2 × SSC at room temperature. Signal amplification was performed by treating the slides with a biotinylated goat anti-avidin (Vector Laboratories) (5 μg/ml in 2 × SSC plus 5% BSA) for 40 min at room temperature. Preparations were washed again three times for 5 min with 2 × SSC and a new incubation with fluorescein avidin D (Vector Laboratories) were carried out for 40 min at room temperature. The slides were counterstained with 0.5 μg/ml DAPI in 2 × SSC solution for 5 min, rinsed in 2 × SSC and mounted in Citifluor antifade solution (Citifluor Ltd, UK).

### Microscopy and Image Acquisition

For image acquisition the confocal microscope Leica TCS SP5 equipped with immersion 100× objective, 488 nm argon and 405 nm diode lasers was used. For primary image analysis Leica LAS AF software was used. The series of confocal sections were collected with the step size 0.25 μm, and maximal projections of the series were obtained. Negative (inverse) DAPI-banding pattern that is coincided with G-banding one was computer processed according to the protocol published [84]. Chromosome identification was going on with the help of images of individual G-banded mouse chromosomes with different level of compaction [41].

## Authors' contributions

GEV and DSJ performed probe labelling, FISH and chromosome identification. KAS performed genomic analysis and probe design. IAM participated in data interpretation and helped to draft the manuscript. POI and KAS conceived the experimental design, analyzed and interpreted data. POI wrote the final manuscript and coordinated the project. All authors have read and approved the final manuscript.

## Supplementary Material

Additional file 1 **Supplementary tables**. This file can be viewed with: Adobe Acrobat Reader. Click here for file

Additional file 2 **Coordinates of MaSat arrays**. This file can be viewed with: Adobe Acrobat Reader. Click here for file

Additional file 3 **Supplementary figures**. This file can be viewed with: Adobe Acrobat Reader. Click here for file
