# Supplementary material for: Tandemly repeated DNA families in the mouse genome
Source: BMC Genomics. 2011 Oct 28;12:531. doi: 10.1186/1471-2164-12-531 (PMC3218096; doi:10.1186/1471-2164-12-531)
Supplement: Additional file 1 — Supplementary tables. This file can be viewed with: Adobe Acrobat Reader. [file 1471-2164-12-531-S1.PDF]

Additional file 1 – Tables S1-S7

Table S1 – Distances from CEN gap to a first gene in the reference genome (build 37.1)

For each chromosome except Y (Chr) distances from a first gene to centromeric gap (Dist), a number of genes (N genes) and a position of first TR array (Dist TR, "-" – not found, "0" – TR array is located just at the gap) in the 2 Mb region adjusted to centromeric gap are shown. Gene ID and type of the evidences are shown.

Chr	Dist (kb)	N genes	Dist TR (kb)	First gene	Gene ID	Gene evidence type
1	205	17	-	Xkr4	497097	best RefSeq; identical
2	36	31	-	9630050M13Rik	269233	best RefSeq; identical
3	149	9	0	Trnak-cuu	100093675	External
4	13	23	6	LOC100039124	100039124	Protein
5	0	26	-	LOC171266	171266	best RefSeq; mismatch
6	19	22	82	LOC100039885	100039885	Protein
7	72	91	-	LOC100041028	100041028	Protein
8	83	62	-	EG664801	664801	Protein
9	134	16	0	4930433N12Rik	114673	mRNA
10	134	11	-	Cnksr3	215748	best RefSeq; identical
11	19	58	0	LOC236604	236604	best RefSeq
12	13	36	-	LOC668260	668260	Protein
13	8	30	-	LOC667375	667375	mRNA
14	4	68	-	LOC100040452	100040452	mRNA; identical
15	3	15	-	LOC674207	674207	mRNA
16	99	47	232	LOC100042177	100042177	mRNA; identical
17	42	20	6	LOC383196	383196	protein; identical
18	161	20	113	LOC664794	664794	Protein
19	94	19	-	LOC630980	630980	Protein
X	32	31	-	LOC385516	385516	best RefSeq

Table S2 – WGS MiSat arrays with length >3 kb
For each array found in WGS row index (N), unit length (Unit), array length (Length), GC%, variability between monomers in array (Var%) and GenBank GI (GI) with array position (Start and End pos) are shown.

N	Unit (bp)	Length (bp)	GC%	Var%	GI	Start pos	End pos
1	112	3105	33.1	7	69824452	1	3105
2	112	3054	33.2	9	69824279	1	3054
3	112	3035	33.1	6	69824189	1	3035
4	112	3020	33.0	7	69824129	1	3020
5	112	3614	33.3	10	69825764	1	3614
6	112	3505	32.6	8	69825514	1	3505
7	112	3376	32.6	8	69825237	1	3376
8	112	3255	32.8	9	69824919	1	3255
9	112	3171	33.3	15	69824647	1	3171
10	120	3850	31.7	17	69780418	956	4805
11	120	3029	33.1	5	69824173	1	3029
12	120	6080	32.2	15	69778468	1	6080
13	120	3142	32.9	18	69825085	177	3318
14	223	3874	31.7	16	69776774	1	3874
15	232	4672	31.4	15	69827328	1	4672
16	232	4670	31.6	15	69777779	1	4670
17	232	3434	31.2	16	69825410	19	3452
18	232	5491	31.6	12	69828178	1	5491
19	240	4490	32.2	15	69827114	8	4497
20	360	4694	32.4	14	69827298	1	4694
21	1054	3206	31.9	10	69824761	1	3206

Table S3– WGS Multi locus, Single locus and Unplaced TR arrays with length >3 kb

Columns names are the same as in Table S3 with the subfamily name added for each array.

N	Subfamily	Unit (bp)	Length (bp)	GC%	Var%	NCBI GI	Start pos	End pos
				**TR-21A-MM**				
1	TRPC-21A-MM	21	4869	49	28	69913686	12	4880
2	TRPC-21A-MM	21	4302	51	29	69885816	2279	6580
3	TRPC-21A-MM	21	15684	50	28	69845347	2865	18548
4	TRPC-21A-MM	21	10656	50	27	69845346	1	10656
5	TRPC-21A-MM	21	3336	51	27	69825142	1	3336
6	TRPC-21A-MM	21	6763	49	30	69551604	2806	9568
7	TRPC-21A-MM	21	3775	50	29	69829807	4	3778
8	TRPC-21A-MM	21	7726	48	31	20779795	3	7728
9	TRPC-21A-MM	21	4581	50	27	69827752	481	5061
10	TRPC-21A-MM	21	11791	50	32	69787202	12893	24683
11	TRPC-21A-MM	21	3120	50	31	20595812	73097	76216
12	TRPC-21A-MM	21	4956	49	30	20595819	1405	6360
13	TRPC-21A-MM	21	4089	48	30	20595822	2	4090
14	TRPC-21A-MM	21	4349	48	31	20595828	296	4644
15	TRPC-21A-MM	21	5078	50	27	20615795	3	5080
16	TRPC-21A-MM	21	5288	48	30	20629104	4626	9913
17	TRPC-21A-MM	21	3152	49	29	20646974	47	3198
18	TRPC-21A-MM	21	3430	50	31	20653070	4	3433
19	TRPC-21A-MM	21	4335	49	31	20663797	124	4458
20	TRPC-21A-MM	21	3584	49	29	20663806	1	3584
21	TRPC-21A-MM	21	4101	49	27	20679820	14184	18284
22	TRPC-21A-MM	21	3584	48	30	20698680	10019	13602
23	TRPC-21A-MM	21	4787	49	31	20716982	22	4808
24	TRPC-21A-MM	21	3221	51	28	20721109	3	3223
25	TRPC-21A-MM	21	14741	50	28	20725869	1	14741
26	TRPC-21A-MM	21	12118	50	28	20726665	27	12144
27	TRPC-21A-MM	21	7698	49	32	20733427	3	7700
28	TRPC-21A-MM	21	7307	48	31	69798865	2	7308
29	TRPC-21A-MM	21	5903	49	27	69798864	1	5903
30	TRPC-21A-MM	21	8021	49	29	69798860	9	8029
31	TRPC-21A-MM	21	3715	49	29	69798859	66	3780
32	TRPC-21A-MM	21	4107	50	31	20757956	2436	6542
33	TRPC-21A-MM	21	6271	49	28	69798688	11593	17863
34	TRPC-21A-MM	21	9932	50	27	20763719	1	9932
35	TRPC-21A-MM	42	29884	48	33	69970350	142	30025
36	TRPC-21A-MM	42	7307	49	30	20779788	9	7315
37	TRPC-21A-MM	42	15628	50	30	69779856	8	15635
38	TRPC-21A-MM	42	7426	48	30	69779230	703	8128

N	Subfamily	Unit (bp)	Length (bp)	GC%	Var%	NCBI GI	Start pos	End pos
39	TRPC-21A-MM	42	4739	49	27	20706577	228	4966
40	TRPC-21A-MM	42	3738	48	27	20725369	218	3955
41	TRPC-21A-MM	42	6167	48	30	69798871	2099	8265
42	TRPC-21A-MM	63	4417	50	28	69845345	3	4419
43	TRPC-21A-MM	63	8397	48	32	69970350	58294	66690
44	TRPC-21A-MM	63	12594	50	28	69779062	4	12597
45	TRPC-21A-MM	63	4386	49	30	69798870	1	4386
46	TRPC-21A-MM	125	3137	49	32	20725866	4	3140
47	TRPC-21A-MM	168	10758	49	32	69970349	50388	61145
48	TRPC-21A-MM	209	17198	49	31	69970350	33180	50377
49	TRPC-21A-MM	209	9587	48	32	20698680	294	9880
50	TRPC-21A-MM	209	7481	48	29	69798863	17	7497
			**Multi locus TR**					
51	TR-4A-MM	4	7704	44	37	69762187	10027	17730
52	TR-4A-MM	6	3001	28	29	69760073	30856	33856
53	TR-4A-MM	8	3063	37	30	20741311	1472	4534
54	TR-4A-MM	10	3416	27	34	69760073	36036	39451
55	TR-4A-MM	19	3304	32	31	69895613	541	3844
56	TR-4A-MM	23	3661	30	18	69995841	18009	21669
57	TR-4B-MM	4	4161	44	38	69762184	3574	7734
58	TR-4B-MM	30	3294	48	33	69798594	275	3568
59	TR-6A-MM	6	4151	60	40	69772082	12	4162
60	TR-6A-MM	14	6649	60	39	69772081	68	6716
61	TR-16A-MM	16	6765	53	24	69920266	4719	11483
62	TR-16A-MM	16	3350	55	11	69894355	7221	10570
63	TR-18A-MM	19	4013	56	25	69770893	9	4021
64	TR-18A-MM	19	5644	56	29	20615423	2663	8306
65	TR-18A-MM	19	3855	55	28	69780122	4397	8251
66	TR-19B-MM	19	4175	46	13	69874805	8073	12247
67	TR-19B-MM	19	5852	51	30	20692411	12070	17921
68	TR-19B-MM	19	4062	46	14	20740135	1	4062
69	TR-20A-MM	20	3470	55	26	69767056	338141	341610
70	TR-20A-MM	20	3604	36	36	69980749	39780	43383
71	TR-22A-MM	22	4784	58	23	69924996	3752	8535
72	TR-22A-MM	22	3429	59	30	69777571	16	3444
73	TR-22A-MM	22	8198	58	23	69916504	11	8208
74	TR-22A-MM	22	5824	58	23	69916503	5	5828
75	TR-22A-MM	22	3090	59	25	69863943	4	3093
76	TR-22A-MM	22	5424	59	25	69863942	2	5425
77	TR-22A-MM	22	12896	57	23	69780624	1	12896
78	TR-22A-MM	22	5674	57	22	69798385	3	5676
79	TR-22A-MM	44	8522	58	27	69916502	34698	43219
80	TR-27A-MM	27	3346	39	26	69892976	4096	7441
81	TR-27A-MM	27	7073	39	29	69863967	1	7073
82	TR-27A-MM	27	4261	40	26	69863966	15	4275

N	Subfamily	Unit (bp)	Length (bp)	GC%	Var%	NCBI GI	Start pos	End pos
83	TR-27A-MM	27	4929	40	26	20735549	1	4929
84	TR-30A-MM	30	3912	46	37	69844207	316	4227
85	TR-31A-MM	31	3673	50	13	69826951	740	4412
86	TR-31A-MM	93	6558	52	27	69864994	1866	8423
87	TR-31B-MM	31	3037	53	18	69778775	1	3037
88	TR-31B-MM	31	4922	53	20	69778774	1	4922
89	TR-31C-MM	31	3147	50	23	69406430	1	3147
90	TR-31C-MM	31	13059	49	25	69963525	1	13059
91	TR-31C-MM	31	3584	49	23	69963521	1	3584
92	TR-31C-MM	31	10181	50	25	20654307	3	10183
93	TR-38C-MM	38	6796	50	26	69871294	6158	12953
94	TR-38C-MM	38	6797	50	29	20595065	37970	44766
95	TR-38C-MM	38	3601	46	22	20622731	26471	30071
96	TR-57A-MM	57	3229	59	30	69887864	83720	86948
97	TR-57A-MM	192	3228	59	31	69887864	83719	86946
98	TR-57A-MM	1869	5619	39	1	20614333	30335	35953
99	TR-58A-MM	58	4652	51	34	69922432	22463	27114
100	TR-58A-MM	58	4658	51	35	20611377	1479	6136
101	TR-81A-MM	81	3483	40	31	69845344	11316	14798
102	TR-1149A-MM	1149	5463	46	13	69830128	1816	7278
103	TR-1164A-MM	1164	3333	49	19	69763564	3174	6506
104	TR-1521A-MM	1521	3211	45	11	20694878	25015	28225
105	TR-1521A-MM	1526	3213	45	12	69980699	1682	4894
106	TR-1527A-MM	1527	3120	46	12	20695066	1	3120
107	TR-1595A-MM	1595	3007	39	16	69766272	1407	4413
				**Single locus TR**				
108	TR-17A-MM	17	3748	44	31	69842800	104362	108109
109	TR-17A-MM	17	3443	44	31	20724788	12	3454
110	TR-17A-MM	38	10270	43	34	69842800	78622	88891
111	TR-17A-MM	75	10312	43	35	69842800	78568	88879
112	TR-17A-MM	173	3828	43	34	20735817	16645	20472
113	TR-19A-MM	19	4883	50	32	69839474	1	4883
114	TR-19A-MM	19	4101	50	32	20610023	100	4200
115	TR-23A-MM	23	6018	43	13	20784691	54	6071
116	TR-24C-MM	24	3298	53	9	69873212	8626	11923
117	TR-29A-MM	29	4175	51	27	20782309	21613	25787
118	TR-29A-MM	29	3389	50	26	20567260	3	3391
119	TR-29A-MM	29	3540	50	25	20567262	1329	4868
120	TR-29A-MM	145	3591	50	28	20567262	749	4339
121	TR-29B-MM	29	15896	50	30	69974133	17596	33491
122	TR-31D-MM	31	5175	56	24	69827874	1	5175
123	TR-33A-MM	33	3601	53	17	20584741	3	3603
124	TR-34A-MM	34	3352	55	7	69872454	20569	23920
125	TR-34A-MM	34	3354	55	7	20680935	20521	23874
126	TR-38A-MM	38	5426	42	27	20783783	76	5501

N	Subfamily	Unit (bp)	Length (bp)	GC%	Var%	NCBI GI	Start pos	End pos
127	TR-38A-MM	38	3941	44	23	20670356	6	3946
128	TR-38B-MM	38	6113	42	26	69904150	1	6113
129	TR-38B-MM	38	4436	42	25	69904117	1	4436
130	TR-39A-MM	39	3387	39	5	20793760	481	3867
131	TR-40A-MM	40	6612	63	21	69856745	14026	20637
132	TR-44A-MM	44	3016	36	9	20625740	1	3016
133	TR-48A-MM	48	6603	51	28	69861320	12400	19002
134	TR-54A-MM	54	4947	48	28	20687861	2718	7664
135	TR-54A-MM	162	10744	48	32	20752452	3470	14213
136	TR-54A-MM	269	5702	48	35	20756725	1	5702
137	TR-54B-MM	54	3164	48	26	69768762	6466	9629
138	TR-54B-MM	108	11978	48	33	69768777	40749	52726
139	TR-54B-MM	161	3322	48	28	69768758	369	3690
140	TR-54B-MM	162	10870	48	31	69768762	6494	17363
141	TR-56A-MM	56	4194	43	22	69768250	2137	6330
142	TR-56A-MM	168	4046	42	23	69768246	2003	6048
143	TR-84A-MM	84	3040	49	9	20793506	14440	17479
144	TR-93A-MM	93	3124	51	24	69825195	16	3139
145	TR-100A-MM	100	4364	44	26	69768907	7155	11518
146	TR-100A-MM	100	4364	44	26	20680076	7577	11940
147	TR-111A-MM	111	3347	52	22	20782355	1996	5342
148	TR-234A-MM	234	4472	60	4	20792971	2	4473
149	TR-234A-MM	696	6878	59	6	20794729	16026	22903
150	TR-297A-MM	297	3100	56	19	69842306	98123	101222
151	TR-321A-MM	321	3152	42	21	20643323	25711	28862
152	TR-734A-MM	734	9507	37	24	20747431	35163	44669
153	TR-734A-MM	740	8739	37	21	20747431	26230	34968
154	TR-734A-MM	1474	4899	37	21	69762220	13783	18681
155	TR-814A-MM	814	3175	45	3	20649433	1845	5019
156	TR-1146A-MM	1146	3056	47	14	69834409	63293	66348
157	TR-1284A-MM	1284	5239	42	14	69888739	38050	43288
158	TR-1384A-MM	1384	3665	26	8	20679591	308	3972
159	TR-1870A-MM	1870	5795	45	2	20649296	1	5795
160	TR-1870A-MM	1872	4818	44	5	20646096	1	4818
161	TR-1870A-MM	1873	4540	45	3	20675563	1	4540
162	TR-1872A-MM	1872	4285	43	17	69899672	10072	14356
163	TR-1908A-MM	1908	4126	40	14	20745884	6859	10984
				**Unplaced TR**				
164	TR-13A-MM	13	4477	56	15	69917804	1327	5803
165	TR-24A-MM	24	3322	46	23	20641441	6	3327
166	TR-24A-MM	24	3540	45	22	20756212	1	3540
167	TR-24B-MM	24	4393	35	26	69883785	2	4394
168	TR-24B-MM	24	7636	34	28	69883682	2	7637
169	TR-24B-MM	24	4707	34	29	69827337	1	4707
170	TR-27B-MM	27	3452	64	36	69798693	7952	11403

N	Subfamily	Unit (bp)	Length (bp)	GC%	Var%	NCBI GI	Start pos	End pos
171	TR-28A-MM	28	3195	41	23	69825025	1	3195
172	TR-36A-MM	36	3003	64	8	69834529	8	3010
173	TR-102A-MM	102	3698	46	31	69765558	1	3698
174	TR-624A-MM	624	3297	43	2	20754462	1	3297

Table S4 – WGS TE-related arrays with length >3 kb
Columns names are the same as in Table S3.

N	Unit (bp)	Length (bp)	GC%	Var%	GI	Start pos	End pos
				**TR-MTA-MM**			
1	1478	3351	45.8	1	20568724	10446	13796
2	1484	3363	46.2	1	69867327	15344	18706
3	1488	3225	45.5	19	69762556	22864	26088
4	1488	3379	45.4	18	20681598	1809	5187
5	1489	4850	45.4	1	69900412	13860	18709
6	1490	3375	45.8	0	69916732	23310	26684
7	1491	3370	45.9	2	69921411	13359	16728
8	1492	3381	45.2	0	69866223	1031	4411
9	1493	3497	45.4	3	20676965	258	3754
10	1493	3377	45.8	2	20705703	6907	10283
11	1494	3386	46.3	1	69958941	35314	38699
12	1497	3384	45.1	2	20610870	2687	6070
13	1502	4272	46.4	1	20583185	1	4272
14	1505	3352	46.1	2	20564801	17230	20581
15	1551	3498	45.0	4	20637905	13405	16902
				**TR-LINE-MM**			
16	1090	3284	42.9	9	20565614	5144	8427
17	1119	3127	41.7	11	20613371	2668	5794
18	1185	3559	39.6	5	20698228	1682	5240
19	1304	3880	44.7	4	69956417	1350	5229
20	1528	3973	40.5	5	69762966	24672	28644
21	1559	3114	42.0	17	69834101	27784	30897
22	1581	3169	42.4	8	69983060	25193	28361
23	1590	3187	43.8	3	69837416	15306	18492
24	1595	3195	42.6	12	20778512	12941	16135
25	1601	3251	43.1	7	20701771	2788	6038
26	1627	4214	42.3	3	20600012	272	4485
27	1874	3712	42.2	10	69834759	16426	20137
28	1877	3702	42.1	11	20597462	22	3723
29	1962	3863	42.0	9	69885494	34804	38666
30	1982	3949	42.4	9	69960655	25917	29865

Table S5 – Positions of TE-related arrays with length >3 kb in the mouse reference genome (build 37.1)

For each array row index (N), chromosome N (Chr) and Chromo band, start and end position in the reference genome are shown. Alignment length is the length of a genomic region covered with precise array.

N	Chr	Chromo Band	Start Position (bp)	End Position (bp)	Alignment length (bp)
			**TR-LINE-MM**		
1	1	1A2	6538348	6541607	3259
2	1	1B	26546515	26550550	4035
3	1	1B	30527087	30530065	2978
4	1	1C1.2	44531737	44535648	3911
5	1	1C1.2	46498285	46501441	3156
6	1	1C1.2	46756918	46760143	3230
7	1	1C1.2	48744898	48748065	3167
8	1	1C1.2	48910328	48914008	3743
9	1	1C4	67764812	67768002	3194
10	1	1C4	70007668	70010917	3249
11	1	1D	81444956	81448665	3790
12	1	1E1.2	100291932	100294973	3041
13	1	1E1.2	101437680	101440834	3225
14	1	1E1.2	101474628	101477508	2880
15	1	1E1.2	101821917	101825051	3147
16	1	1E2.2	108570242	108573341	3099
17	1	1E2.2	111152468	111155646	3843
18	1	1E3	114267839	114271625	3786
19	1	1E3	115617472	115620725	3266
20	1	1E3	119585097	119588204	3108
21	1	1F	132301392	132304559	3167
22	1	1G2	148889162	148892240	3078
23	1	1H5	179759633	179762784	3151
24	1	1H5	179847620	179851576	3956
25	2	2A2	3764054	3767782	3811
26	2	2A2	11912285	11915749	3464
27	2	2A2	12584733	12588362	3629
28	2	2B	15502152	15505315	3185
29	2	2B	17011062	17014036	2974
30	2	2C1.2	48906504	48909523	3019
31	2	2C1.2	56784083	56787377	3294
32	2	2C1.2	57424863	57428488	3708
33	2	2E2	83218105	83221691	3586
34	2	2E2	86588263	86591281	3018
35	2	2E2	87724158	87727847	3752
36	2	2E2	95055992	95059259	3267
37	2	2E2	95125623	95128884	3261
38	2	2E2	97683248	97686487	3245
39	2	2E4	111012787	111015776	2989
40	2	2F3	123772834	123776340	3506
41	2	2G2	139086653	139089811	3158
42	2	2G2	140704524	140707406	2882
43	2	2H1	151277926	151281284	3358
44	2	2H1	151303993	151307247	3254
45	3	3A2	4329803	4332693	2890
46	3	3A2	5112053	5115147	3099
47	3	3A2	6036134	6039150	3016
48	3	3A2	6599986	6603129	3143
49	3	3A2	10002125	10005358	3233
50	3	3A2	18252262	18256254	3998
51	3	3B	18882173	18885328	3159

N	Chr	Chromo Band	Start Position (bp)	End Position (bp)	Alignment length (bp)
		**TR-LINE-MM**			
52	3	3B	22540031	22544101	4083
53	3	3B	22883572	22886468	2896
54	3	3D	41650908	41653916	3019
55	3	3D	55944653	55947734	3081
56	3	3E2	61475717	61478708	2991
57	3	3E2	61930403	61934298	3895
58	3	3F1	70411095	70414545	3450
59	3	3F1	81397986	81401269	3283
60	3	3F2.2	90707536	90710613	3077
61	3	3F3	106663642	106667609	3971
62	3	3H1	133515183	133519087	3904
63	3	3H1	134732641	134735786	3145
64	3	3H3	139038573	139041743	3170
65	3	3H3	144433609	144436861	3252
66	4	4A2	4200374	4203694	3320
67	4	4A2	7634501	7638447	3949
68	4	4A4	18992723	18996641	3918
69	4	4C4	74748540	74752240	3700
70	4	4C4	80127224	80130418	3223
71	4	4C6	89385628	89388853	3225
72	4	4C6	96512963	96516768	3876
73	4	4D2.2	113513641	113516819	3184
74	4	4D2.2	114981068	114984491	3423
75	5	5A2	12776311	12779975	3664
76	5	5B1	15045395	15048626	3255
77	5	5B3	26634125	26637339	3218
78	5	5C2	54950922	54954121	3228
79	5	5E2	85538841	85541710	2869
80	5	5E2	89762491	89766353	3862
81	5	5E4	94054232	94057403	3171
82	6	6B3	49835162	49838351	3189
83	6	6C2	65247127	65256435	9330
84	6	6C2	69505679	69508678	2999
85	6	6C2	73495770	73499723	3953
86	6	6D3	89679335	89682498	3168
87	6	6D3	90169142	90172227	3085
88	6	6F3	121879755	121883404	3649
89	6	6F3	123080116	123084000	3955
90	6	6G2	131718311	131721314	3003
91	6	6G2	132585280	132588477	3226
92	6	6G3	142127405	142131316	3914
93	7	7B1	17811407	17814548	3154
94	7	7B3	32170794	32173801	3007
95	7	7B3	32906624	32909783	3159
96	7	7B3	33645188	33648351	3163
97	7	7B3	33872085	33875247	3162
98	7	7B3	39125037	39128802	3765
99	7	7B5	48965411	48968513	3102
100	7	7B5	55826053	55829745	3692
101	7	7D1	62858952	62862142	3202
102	7	7D1	68100912	68104131	3230
103	7	7D1	68251632	68254828	3210
104	7	7D1	70544436	70547294	2858
105	7	7E2	95824993	95828113	3132
106	7	7E2	96842603	96845823	3246
107	7	7F1	113541782	113545673	3891
108	8	8A4	30621163	30624226	3063
109	8	8B3.2	58668811	58671827	3016

N	Chr	Chromo Band	Start Position (bp)	End Position (bp)	Alignment length (bp)
			**TR-LINE-MM**		
110	8	8B3.2	58834024	58837215	3197
111	8	8B3.2	62708560	62711735	3180
112	8	8B3.2	64506155	64509542	3387
113	8	8B3.2	65774983	65780527	5607
114	8	8B3.2	66298633	66302587	3954
115	8	8C1	69049720	69053257	3537
116	8	8C1	73574835	73578831	3996
117	8	8D2	101192544	101195583	3039
118	8	8E1	105470284	105473252	2968
119	8	11A2	131299974	131304452	4478
120	9	9A2	8229749	8232922	3173
121	9	9A2	12538018	12541977	3963
122	9	9A4	16599046	16602975	3929
123	9	9A4	18043919	18047872	3953
124	9	9A4	23464563	23468525	3962
125	9	9A5.2	25649201	25652183	2982
126	9	9A5.2	25979682	25982733	3051
127	9	9A5.2	26680326	26683552	3226
128	9	9E3.2	83172562	83176224	3745
129	9	9E3.2	84049846	84052779	2933
130	9	9E3.2	87684471	87689308	4840
131	9	9F3	105048812	105051983	3171
132	9	9F3	109204212	109208033	3824
133	9	9F3	109256192	109260011	3890
134	9	9F3	109295944	109299170	3230
135	10	10B2	38479057	38482588	3531
136	10	10B4	47248386	47251665	3280
137	10	10B4	47615536	47618790	3254
138	10	10B4	48314097	48317981	3955
139	10	10B4	49560360	49563567	3207
140	10	10B4	49657259	49660237	2978
141	10	10B4	52214918	52218602	3747
142	10	10B5.2	65787396	65791114	3807
143	10	10C3	95467930	95471050	3120
144	10	10D2	105387660	105390703	3043
145	11	11A2	8222737	8226666	3933
146	11	11A2	9256588	9259930	3342
147	11	11A2	11993516	11996414	2898
148	11	11A3.2	13763035	13766717	3787
149	11	11A3.2	16281623	16285264	3705
150	11	11A4	26758967	26762451	3484
151	11	11A4	28685025	28688181	3156
152	11	11A4	29236183	29239481	3298
153	11	11B1.1	40055348	40058248	2971
154	11	11B1.3	45420129	45423055	2926
155	11	11D	89826761	89829962	3210
156	11	11E2	105565019	105568704	3748
157	12	12A1.2	5803240	5806391	3151
158	12	12A1.2	9784395	9788057	3662
159	12	12B1	27926337	27929757	3420
160	12	12C2	45589211	45593128	3921
161	12	12C2	50958842	50961981	3139
162	12	12C2	62303932	62307122	3235
163	12	12C2	65862526	65866307	3852
164	12	12F1	102471912	102475082	3199
165	12	12A1.2	115950311	115954025	3841
166	12	12C2	116121534	116125302	3839
167	12	12F1	116890059	116892978	2922

N	Chr	Chromo Band	Start Position (bp)	End Position (bp)	Alignment length (bp)
		**TR-LINE-MM**			
168	12	12A1.2	116983962	116986931	2969
169	13	13A2	11476000	11479490	3490
170	13	13A3.2	16342821	16346681	3860
171	13	13A3.2	22929568	22932637	3069
172	13	13B1	50591334	50595305	3976
173	13	13B3	61329896	61332956	3060
174	13	13C2	70015945	70019825	3880
175	13	13C2	77841875	77845077	3212
176	13	13D1	82157765	82160938	3178
177	13	13D1	84979160	84982082	2922
178	13	13D1	87326313	87329409	3096
179	13	13D1	90587629	90591585	3956
180	13	13D1	92089603	92092909	3327
181	14	14C2	37193667	37197484	3817
182	14	14C2	42679815	42683634	3819
183	14	14C2	43024244	43028063	3819
184	14	14C2	43438020	43441839	3819
185	14	14C2	43629218	43633038	3820
186	14	14D1	59501748	59505477	3729
187	14	14D3	71456588	71459633	3045
188	14	14E5	109969382	109972543	3161
189	14	14E5	119637177	119640212	3035
190	15	15A2	5323741	5327600	3863
191	15	15A2	14889788	14892765	2977
192	15	15B2	18590028	18594015	3987
193	15	15B2	19064719	19068627	3911
194	15	15B2	20435500	20438676	3176
195	15	15B2	24735827	24738987	3164
196	15	15B2	26099537	26103433	3896
197	15	15B2	29275259	29278377	3123
198	15	15B3.2	29924994	29928191	3226
199	15	15B3.2	33658595	33662420	3896
200	15	15B3.2	39526886	39530869	3983
201	15	15C	45634175	45637441	3297
202	15	15D2	52353886	52357074	3217
203	15	15D2	63626140	63629326	3191
204	15	15D2	65894286	65897450	3187
205	15	15E3	82554452	82557507	3060
206	17	17B3	38320865	38324574	3772
207	17	17B3	38375792	38379483	3755
208	17	17B3	92821587	92825279	3755
209	18	18A2	12484457	12487579	3122
210	18	18A2	19195065	19197960	2895
211	18	18C	41674103	41677391	3288
212	18	18C	41763448	41766618	3170
213	18	18D2	50597051	50600235	3184
214	18	18D2	52612627	52615699	3072
215	18	18E1	60498102	60502037	3935
216	18	18E1	85786652	85790379	3727
217	18	18C	90224992	90228683	3773
218	19	19B	7993081	7996231	3150
219	19	19B	9726672	9730270	3598
220	19	19B	13323411	13326359	2948
221	19	19B	14045002	14048115	3113
222	19	19D1	39426481	39432408	5927
223	19	19D3	51728435	51732398	3963
224	19	19D3	52505202	52508855	3653
225	X	XA1.2	7933876	7936864	2988

N	Chr	Chromo Band	Start Position (bp)	End Position (bp)	Alignment length (bp)
			**TR-LINE-MM**		
226	X	XA1.2	8055758	8059013	3255
227	X	XA1.2	8361541	8364515	2974
228	X	XA2	19354237	19358030	3796
229	X	XA2	19621522	19624644	3140
230	X	XA3.2	21100522	21104454	3932
231	X	XA3.2	22606305	22610003	3761
232	X	XA4	36184421	36187755	3334
233	X	XA4	36967814	36970874	3060
234	X	XA6	47255840	47258912	3072
235	X	XA7.2	61138059	61141954	3966
236	X	XA7.2	61162816	61165732	2916
237	X	XA7.2	63588958	63592482	3527
238	X	XA7.2	67525370	67528424	3054
239	X	XB	68591885	68594881	2996
240	X	XB	72603481	72607492	4011
241	X	XB	73798138	73801477	3339
242	X	XC2	82617192	82620235	3043
243	X	XD	90488309	90491966	3740
244	X	XD	92461140	92465114	3974
245	X	XE2	105690272	105693332	3060
246	X	XE2	105804998	105807945	2947
247	X	XE2	112647960	112650875	2915
248	X	XE2	116796602	116800031	3429
249	X	XF1	123360830	123364018	3192
250	X	XF1	125021740	125024862	3122
251	X	XF1	125847938	125850879	2941
252	X	XF3	132164562	132167756	3199
253	X	XF3	133950754	133954654	3904
254	X	XF3	138583415	138587051	3714
255	X	XF3	142144293	142148029	3799
256	X	XF5	146806123	146809930	3810
257	X	XF5	151446178	151449374	3204
258	X	XF5	153758165	153761282	3132
259	X	XF5	155336215	155339255	3040
260	X	XF5	155571523	155576301	4801
261	X	XF5	162092385	162095448	3063
262	X	XF5	165195974	165198845	2900
			**TR-MTA-MM**		
263	1	1C1.2	32014240	32022623	8383
264	2	2A2	5466924	5469925	3001
265	2	2B	20344705	20348204	3499
266	2	2C1.2	46198663	46203548	4885
267	3	3F1	77771511	77774895	3385
268	3	3F2.2	96074241	96077418	3177
269	4	4B1	37970056	37973441	3385
270	4	4C6	86104230	86109095	4865
271	5	5F	103128255	103131638	3383
272	6	6E2	102800300	102803770	3470
273	10	19D3	129900158	129903529	3371
274	12	12C2	52134210	52137613	3403
275	12	12D3	84388876	84392397	3521
276	12	12D3	84657947	84661468	3521
277	16	16C1.2	44947448	44950829	3381
278	16	16C4	85781779	85785167	3388
279	17	17B3	33191637	33195008	3371
280	17	17B3	34599239	34602615	3378
281	19	19D1	39330195	39345529	15334
282	19	19D3	53042426	53045797	3371

N	Chr	Chromo Band	Start Position (bp)	End Position (bp)	Alignment length (bp)
			**TR-LINE-MM**		
283	X	XB	69435096	69438444	3350
284	X	XC2	77211342	77214715	3373

Table S6 – Probes used for FISH

For each probe, subfamily and family (according to the tables 4-6), probe type: double stranded (DS) or single stranded (SS), probe length with monomer multiplication, and probe sequence are shown. In the sequences every ten nucleotide is in bold, adaptors indicated by upper case.

N	Subfamily	Family	Type	Length	Sequence
1	TRPC-21A-MM	C3, ML	DS	163 bp<br>(42 bp x3)	GGTCGAAGA**C**ACGAAGAAC**T**TTtgtcacag**t**gctccgctgt**g**gtgacaaag**t**<br>gtctactg**t**gttgcaaag**t**gctcaactt**t**tgtgtcaca**a**tgctgcact**g**tgttgtcac**a**ttctggaac**t**<br>gtggtgtca**c**agtgttcca**c**tttttAGAC**C**GTCATCGGC**G**TAG
2	TRPC-21A-MM	C3, ML	SS	42 bp<br>(21 bp x2)	gtgtcacag**t**gctccactg**t**ggtgtcaca**g**tgctccact**g**tg
3	TR-22A-MM	C4, ML	SS	22 bp	tagccccag**g**gcccaaccc**a**tt
4	TR-54B-MM	C5, SL	DS	151 bp<br>(54 bp x2)	GGTCGAAGA**C**ACGAAGAAC**T**TTggattgg**g**ccttactgt**c**ctttgcata**c**<br>cgcaacaca**c**tctgcagct**a**ggatggact**a**agccttact**g**tccttagac**t**gacctacag**c**<br>acaccctgt**a**gctaggata**c**accAGACCG**T**CATCGGCGT**A**G

Table S7 – Tandem repeats in mouse genome assemblies

Assembly	Build	Assembly size (bp)	Number of contigs	TR (total)	% of assembly	TR (>3 kb)
Reference Genome	37.1	2,654,895,218	21	826,028	2.6%	234
Alternate (Celera) Assembly	37.1	2,679,921,514	21	760,414	2.2%	121
MGSC Assembly	3	2,580,596,378	20*	752,199	2.2%	77
ChrUn Ref	37.1	3,350,358	52	1,032	12.2%	29
ChrUn Alt (Celera)	37.1	95,253,641	12,483	28,090	10.3	704
ChrUn MGSC	3	103,946,130	42,254	38,177	9.9%	111

*except Y chromosome
