# Supplementary material for: Tandemly repeated DNA families in the mouse genome
Source: BMC Genomics. 2011 Oct 28;12:531. doi: 10.1186/1471-2164-12-531 (PMC3218096; doi:10.1186/1471-2164-12-531)
Supplement: Additional file 2 — Coordinates of MaSat arrays. This file can be viewed with: Adobe Acrobat Reader. [file 1471-2164-12-531-S2.PDF]

Additional file 2

List of MaSat arrays in the mouse WGS assemblies with length greater than 3 kb
For each array found in WGS unit length (Unit), array length (Length), GC%, variability between monomers in array (Var%), HOR, and GenBank GI (GI) with array position (Start and End pos) are shown. All MaSat arrays present in ChrUn.

N	Unit (bp)	Length (bp)	GC%	Var%	HOR	GI	Start pos	End pos
1	30	3234	36.5	46	+	69778673	1634	4867
2	58	7262	35.0	31	+	69829786	34	7295
3	58	7942	35.4	31	+	69829517	71	8012
4	58	7563	35.1	31	+	69829356	1	7563
5	58	6037	34.2	30	+	69829296	4	6040
6	58	6725	35.3	33	+	69829197	1	6725
7	58	3126	35.3	33	+	69829087	48	3173
8	58	4809	36.1	30	+	69828991	47	4855
9	58	6572	36.0	31	+	69828909	1	6572
10	58	6424	35.7	34	+	69828831	2	6425
11	58	5042	35.4	30	+	69828825	8	5049
12	58	6046	35.4	31	+	69828614	1	6046
13	58	5597	36.1	30	+	69828374	110	5706
14	58	5424	37.1	32	+	69828164	44	5467
15	58	5250	35.2	31	+	69827957	7	5256
16	58	5062	35.4	32	+	69827764	8	5069
17	58	5049	35.6	33	+	69827741	1	5049
18	58	5001	35.9	29	+	69827710	34	5034
19	58	4945	35.4	31	+	69827619	1	4945
20	58	4925	35.6	30	+	69827589	1	4925
21	58	3438	34.1	32	+	69827529	21	3458
22	58	4539	35.9	31	+	69827148	31	4569
23	58	4545	34.6	30	+	69827115	1	4545
24	58	4396	35.5	31	+	69826932	2	4397
25	58	4338	35.0	32	+	69826878	7	4344
26	58	4260	35.6	29	+	69826799	26	4285
27	58	4183	35.8	30	+	69826678	4	4186
28	58	4048	35.8	31	+	69826544	51	4098
29	58	4064	35.2	31	+	69826540	11	4074
30	58	4043	35.8	33	+	69826458	2	4044
31	58	4028	36.1	31	+	69826429	2	4029
32	58	4010	35.8	30	+	69826402	1	4010
33	58	3953	35.5	37	+	69826390	54	4006
34	58	3917	34.9	31	+	69826302	1	3917
35	58	3929	34.9	33	+	69826300	10	3938
36	58	3845	34.6	33	+	69826261	1	3845
37	58	3868	37.9	29	+	69826197	3	3870
38	58	3830	36.0	29	+	69826179	33	3862
39	58	3351	35.0	31	+	69826178	506	3856
40	58	3796	34.6	33	+	69826057	1	3796
41	58	3407	34.9	32	+	69826048	385	3791
42	58	3729	35.3	31	+	69826010	44	3772
43	58	3659	34.0	32	+	69825951	45	3703
44	58	3662	35.3	32	+	69825874	5	3666
45	58	3642	34.9	34	+	69825813	1	3642
46	58	3632	35.9	31	+	69825807	4	3635
47	58	3623	35.3	30	+	69825806	7	3629
48	58	3558	35.2	31	+	69825717	15	3572
49	58	3590	35.5	31	+	69825714	1	3590
50	58	3537	34.9	32	+	69825615	1	3537

N	Unit (bp)	Length (bp)	GC%	Var%	HOR	GI	Start pos	End pos
51	58	3508	35.6	33	+	69825596	4	3511
52	58	3524	35.0	30	+	69825574	1	3524
53	58	3507	35.1	31	+	69825530	5	3511
54	58	3482	35.4	30	+	69825525	28	3509
55	58	3440	35.9	30	+	69825474	34	3473
56	58	3479	34.8	30	+	69825463	2	3480
57	58	3472	36.5	30	+	69825447	1	3472
58	58	3458	35.6	30	+	69825443	4	3461
59	58	3319	38.0	34	+	69825345	3	3321
60	58	3422	35.0	30	+	69825340	1	3422
61	58	3403	35.0	33	+	69825298	1	3403
62	58	3386	35.0	31	+	69825261	1	3386
63	58	3370	35.7	30	+	69825250	2	3371
64	58	3364	35.2	31	+	69825222	9	3372
65	58	3326	35.7	30	+	69825193	1	3326
66	58	3335	35.9	30	+	69825190	1	3335
67	58	3125	35.2	31	+	69825189	228	3352
68	58	3352	36.3	30	+	69825187	5	3356
69	58	3334	35.7	31	+	69825167	1	3334
70	58	3335	35.4	31	+	69825157	1	3335
71	58	3325	35.3	32	+	69825114	5	3329
72	58	3311	34.8	33	+	69825101	6	3316
73	58	3298	35.3	28	+	69825096	20	3317
74	58	3241	35.3	31	+	69825084	55	3295
75	58	3275	34.5	33	+	69825069	6	3280
76	58	3298	34.9	32	+	69825052	13	3310
77	58	3265	35.8	29	+	69825035	38	3302
78	58	3253	37.8	28	+	69824936	10	3262
79	58	3220	35.6	30	+	69824873	21	3240
80	58	3235	36.6	33	+	69824851	1	3235
81	58	3218	34.9	30	+	69824840	14	3231
82	58	3171	35.4	33	+	69824660	3	3173
83	58	3169	36.7	31	+	69824651	2	3170
84	58	3127	35.9	30	+	69824592	1	3127
85	58	3022	35.2	31	+	69824541	109	3130
86	58	3010	36.9	31	+	69824515	34	3043
87	58	3108	34.4	31	+	69824492	6	3113
88	58	3111	35.3	30	+	69824488	4	3114
89	58	3109	35.4	32	+	69824470	1	3109
90	58	3056	35.1	30	+	69824392	20	3075
91	58	3069	35.9	30	+	69824340	1	3069
92	58	3040	35.0	31	+	69824317	25	3064
93	58	3062	35.5	31	+	69824313	1	3062
94	58	3033	34.9	31	+	69824305	5	3037
95	58	3025	35.5	31	+	69824234	1	3025
96	58	3041	34.9	32	+	69824219	1	3041
97	58	3034	35.8	29	+	69824185	1	3034
98	58	3015	35.0	32	+	69824177	6	3020
99	58	3026	35.5	32	+	69824172	3	3028
100	58	3013	36.9	30	+	69824168	13	3025
101	58	7154	36.6	29	+	69809557	6	7159
102	58	3179	35.7	32	+	69809542	2576	5754
103	58	3675	37.2	30	+	69809536	3	3677
104	58	3027	37.4	29	+	69809462	3	3029
105	58	5270	37.3	35	+	69809461	12	5281
106	58	4930	37.1	29	+	69809459	3068	7997
107	58	7891	37.3	30	+	69809439	1	7891
108	58	4371	35.9	30	+	69809132	1	4371

N	Unit (bp)	Length (bp)	GC%	Var%	HOR	GI	Start pos	End pos
109	58	3715	35.1	33	+	69809131	1	3715
110	58	3783	36.1	31	+	69809129	28	3810
111	58	8067	36.0	34	+	69809128	4	8070
112	58	6305	35.6	34	+	69809024	1	6305
113	58	5249	36.6	33	+	69809021	4	5252
114	58	5542	36.9	31	+	69809018	270	5811
115	58	3127	37.4	31	+	69798858	1622	4748
116	58	3489	37.3	28	+	69798855	61	3549
117	58	5084	36.1	30	+	69798845	36	5119
118	58	6407	36.5	32	+	69798839	166	6572
119	58	4609	36.0	33	+	69798833	1	4609
120	58	3211	36.5	29	+	69798770	3	3213
121	58	9717	37.2	25	+	69798696	8	9724
122	58	9581	36.5	36	+	69798472	2	9582
123	58	7373	34.6	32	+	69798357	3	7375
124	58	5595	35.4	32	+	69798352	1	5595
125	58	5428	34.8	35	+	69798332	27	5454
126	58	3940	35.3	32	+	69798323	7	3946
127	58	4826	35.5	31	+	69798153	1	4826
128	58	3565	34.8	33	+	69798151	1	3565
129	58	3846	34.8	31	+	69798043	53	3898
130	58	5200	34.9	33	+	69798042	40	5239
131	58	3061	34.2	37	+	69798041	29	3089
132	58	4259	34.9	31	+	69798039	10	4268
133	58	3099	35.1	32	+	69798014	36	3134
134	58	3251	34.9	32	+	69798013	2	3252
135	58	7133	37.3	32	+	69797954	4	7136
136	58	4184	37.3	28	+	69797945	1	4184
137	58	9971	35.0	34	+	69797933	1	9971
138	58	5392	35.0	32	+	69797914	1	5392
139	58	5150	34.9	31	+	69787705	1	5150
140	58	3689	35.6	30	+	69787579	1	3689
141	58	5575	35.9	30	+	69787539	1	5575
142	58	3582	35.5	32	+	69787538	1	3582
143	58	3927	37.3	29	+	69787495	36	3962
144	58	4184	37.1	32	+	69787493	25	4208
145	58	3384	36.0	32	+	69787468	4	3387
146	58	6026	35.7	31	+	69787440	35	6060
147	58	3451	34.6	30	+	69787437	61	3511
148	58	4081	35.1	31	+	69787436	1	4081
149	58	5833	35.1	31	+	69787412	1578	7410
150	58	5551	34.9	32	+	69787332	1	5551
151	58	4643	36.1	30	+	69787179	12825	17467
152	58	5649	34.7	31	+	69787071	8	5656
153	58	4069	34.2	30	+	69787032	6	4074
154	58	3772	38.2	31	+	69780706	13165	16936
155	58	3900	35.7	31	+	69780432	10	3909
156	58	3340	35.7	30	+	69780430	1	3340
157	58	3962	35.2	32	+	69780289	66	4027
158	58	3635	34.8	33	+	69780288	4	3638
159	58	5224	35.2	32	+	69780281	6	5229
160	58	4415	34.9	31	+	69780280	54	4468
161	58	3147	35.1	31	+	69780253	1	3147
162	58	3882	35.8	33	+	69780251	4	3885
163	58	3042	34.8	33	+	69780163	4	3045
164	58	3725	35.3	31	+	69780131	1	3725
165	58	8091	34.3	32	+	69780126	1388	9478
166	58	7065	35.7	30	+	69780037	10	7074

N	Unit (bp)	Length (bp)	GC%	Var%	HOR	GI	Start pos	End pos
167	58	5846	36.4	32	+	69779747	2215	8060
168	58	5480	35.9	30	+	69779623	1	5480
169	58	3423	36.8	31	+	69779620	8	3430
170	58	3583	35.0	34	+	69779472	27	3609
171	58	5870	38.2	29	+	69779402	1296	7165
172	58	3014	34.6	33	+	69779248	10	3023
173	58	4430	34.7	31	+	69779247	1	4430
174	58	3019	36.0	32	+	69779064	5	3023
175	58	7819	36.1	29	+	69779060	3	7821
176	58	5334	35.6	31	+	69779000	1	5334
177	58	6014	35.4	33	+	69778999	6	6019
178	58	4664	35.5	33	+	69778733	2	4665
179	58	4056	35.4	33	+	69778707	4	4059
180	58	3294	35.0	32	+	69778495	1	3294
181	58	3727	36.3	32	+	69778459	13	3739
182	58	3781	35.1	30	+	69778435	1	3781
183	58	3887	35.1	32	+	69778173	1	3887
184	58	3167	36.2	29	+	69777951	5	3171
185	58	8418	37.1	32	+	69777927	3	8420
186	58	4660	36.8	33	+	69777926	1	4660
187	58	3599	35.3	32	+	69777715	8	3606
188	58	3356	36.1	34	+	69777607	57	3412
189	58	4453	35.8	31	+	69777606	11	4463
190	58	3337	35.7	30	+	69777471	1	3337
191	58	3276	34.5	30	+	69777259	24	3299
192	58	5031	35.8	31	+	69777178	13	5043
193	58	4293	35.1	32	+	69777056	25	4317
194	58	3574	35.9	29	+	69777052	2	3575
195	58	4894	34.9	34	+	69777001	10	4903
196	58	3501	36.3	30	+	69776871	1	3501
197	58	3377	35.2	29	+	69775756	9	3385
198	58	3539	36.1	27	+	69601673	1306	4844
199	58	4148	36.6	32	+	69970351	1	4148
200	58	4496	34.4	33	+	20772172	1	4496
201	58	8325	36.2	31	+	20778062	6	8330
202	58	5026	36.4	29	+	20778115	1	5026
203	58	5427	35.5	31	+	20784542	2	5428
204	58	4061	35.2	30	+	20641030	19	4079
205	58	4283	36.1	33	+	20646310	724	5006
206	58	3551	34.6	32	+	20648551	28	3578
207	58	3002	36.7	33	+	20668347	32	3033
208	58	3011	37.5	32	+	20668393	62	3072
209	58	4128	34.9	28	+	20680103	1	4128
210	58	3748	34.4	36	+	20683179	2	3749
211	58	3919	36.6	29	+	20688657	1279	5197
212	58	4206	36.0	30	+	20713343	8	4213
213	58	3599	36.2	29	+	20719656	9	3607
214	58	4304	35.6	32	+	20722588	2463	6766
215	58	3498	36.6	33	+	20743451	5	3502
216	58	4315	34.6	34	+	20758157	1	4315
217	59	3972	35.6	28	+	69826353	7	3978
218	59	6658	35.7	30	+	69809556	1	6658
219	59	3287	35.4	30	+	69809537	1	3287
220	59	3534	37.0	26	+	69809456	15182	18715
221	59	6615	36.6	33	+	69809141	4	6618
222	59	3763	36.4	34	+	69809139	2970	6732
223	59	5732	36.6	32	+	69809012	2055	7786
224	59	3245	36.5	29	+	69798778	25	3269

N	Unit (bp)	Length (bp)	GC%	Var%	HOR	GI	Start pos	End pos
225	59	3044	36.3	28	+	69798480	7515	10558
226	59	10914	35.9	32	+	69798310	2	10915
227	59	5647	35.9	33	+	69798309	398	6044
228	59	3342	36.7	32	+	69798306	1125	4466
229	59	15822	37.2	31	+	69798036	1505	17326
230	59	7117	37.0	31	+	69798036	19079	26195
231	59	4586	37.8	33	+	69798035	20	4605
232	59	6872	37.3	27	+	69797946	11044	17915
233	59	5950	37.1	28	+	69787559	595	6544
234	59	22913	37.1	33	+	69787526	33	22945
235	59	5943	36.1	31	+	69787455	69	6011
236	59	4080	35.8	29	+	69780673	4	4083
237	59	9295	37.7	32	+	69780673	15134	24428
238	59	5672	38.1	30	+	69778913	1	5672
239	59	9980	36.2	33	+	69778913	5750	15729
240	59	4252	36.7	33	+	69778864	1458	5709
241	59	5080	36.3	30	+	20766889	1	5080
242	59	3423	36.0	31	+	20775050	2745	6167
243	59	3754	36.8	30	+	20787326	23	3776
244	59	7250	36.8	32	+	20787543	15	7264
245	59	3770	37.1	32	+	20641033	1	3770
246	59	6337	37.4	30	+	20647624	16	6352
247	59	10591	36.9	32	+	20649335	33	10623
248	59	3127	36.1	32	+	20667996	21	3147
249	59	3513	37.2	27	+	20668294	1	3513
250	59	3167	35.8	32	+	20693824	15	3181
251	59	3404	35.7	30	+	20694913	27	3430
252	59	4735	36.2	29	+	20721896	60	4794
253	59	4941	37.5	35	+	20727399	1	4941
254	59	5701	36.2	31	+	20735063	8	5708
255	116	5052	35.4	26	+	69827764	1	5052
256	116	3825	35.0	23	+	69827488	34	3858
257	116	4509	35.3	23	+	69827095	5	4513
258	116	4226	35.5	27	+	69826947	3	4228
259	116	3864	35.5	24	+	69826183	1	3864
260	116	3771	35.3	26	+	69826010	1	3771
261	116	3660	35.2	17	-	69825846	1	3660
262	116	3361	35.9	19	+	69825698	223	3583
263	116	3110	35.1	25	+	69824474	1	3110
264	116	4618	35.5	30	+	69809548	4	4621
265	116	6019	35.9	32	+	69809547	6546	12564
266	116	3587	36.5	30	+	69809544	13	3599
267	116	6836	35.0	27	+	69798266	3	6838
268	116	3573	34.4	26	+	69798264	14	3586
269	116	4836	34.8	24	+	69798261	1	4836
270	116	4605	35.7	32	+	69787387	349	4953
271	116	3689	36.8	37	+	69787031	15	3703
272	116	3320	34.3	26	+	69780213	2	3321
273	116	6229	36.1	26	+	69778732	1	6229
274	116	3014	34.9	20	+	69778494	1	3014
275	116	3668	34.8	29	+	69777975	10	3677
276	116	3669	35.8	22	+	69777790	1	3669
277	116	3911	34.6	25	+	20779497	1875	5785
278	116	4781	35.7	34	+	20646310	223	5003
279	116	5855	35.2	26	+	20710918	28	5882
280	116	3164	35.4	21	+	20735384	1	3164
281	117	4057	36.9	33	+	69893283	7163	11219
282	117	6657	36.9	25	+	69885819	1	6657

N	Unit (bp)	Length (bp)	GC%	Var%	HOR	GI	Start pos	End pos
283	117	14395	36.6	30	+	69885813	3	14397
284	117	10039	36.9	27	+	69885812	1	10039
285	117	5741	34.5	22	+	69828408	9	5749
286	117	3893	34.4	25	+	69826593	3	3895
287	117	4091	35.5	24	+	69826542	7	4097
288	117	7666	36.6	29	+	69809557	240	7905
289	117	4347	35.6	30	+	69809549	1925	6271
290	117	3644	35.3	30	+	69809548	8424	12067
291	117	8267	36.6	29	+	69809546	611	8877
292	117	7148	36.6	32	+	69809545	1	7148
293	117	4959	35.6	34	+	69809539	11273	16231
294	117	8647	36.5	29	+	69809533	1	8647
295	117	3388	35.0	39	+	69809450	1398	4785
296	117	5421	37.6	27	+	69809444	8	5428
297	117	3956	36.4	31	+	69809436	813	4768
298	117	6120	35.5	29	+	69809435	1	6120
299	117	3894	36.4	33	+	69809435	6158	10051
300	117	18293	36.7	35	+	69809140	4	18296
301	117	5076	35.6	34	+	69809136	1	5076
302	117	5067	35.0	29	+	69809131	69	5135
303	117	4439	36.5	29	+	69809127	3830	8268
304	117	5140	36.2	29	+	69809020	1177	6316
305	117	8410	36.4	33	+	69809014	15262	23671
306	117	4521	35.6	28	+	69809011	5	4525
307	117	3387	35.9	29	+	69809011	3374	6760
308	117	3765	37.2	34	+	69798842	1004	4768
309	117	6524	35.5	31	+	69798478	1502	8025
310	117	4465	36.3	29	+	69798472	5124	9588
311	117	3821	36.6	31	+	69797996	1	3821
312	117	8028	36.4	27	+	69797946	352	8379
313	117	7016	37.4	34	+	69787770	1	7016
314	117	10457	37.6	30	+	69787562	1	10457
315	117	3937	37.3	34	+	69787559	8103	12039
316	117	22944	37.1	30	+	69787526	1	22944
317	117	3501	35.6	31	+	69787457	17	3517
318	117	10616	35.7	33	+	69780706	3	10618
319	117	4164	35.4	30	+	69780706	7493	11656
320	117	3691	38.2	28	+	69780705	4	3694
321	117	5006	36.2	28	+	69780673	7507	12512
322	117	5922	38.2	27	+	69779402	1305	7226
323	117	3591	35.1	28	+	69778133	73	3663
324	117	5080	34.8	22	+	69777693	3	5082
325	117	7066	36.8	33	+	69776649	346	7411
326	117	3901	37.3	28	+	69546071	1	3901
327	117	4332	36.2	32	+	69970351	2369	6700
328	117	3315	36.3	36	+	20778080	1	3315
329	117	8975	37.4	27	+	20778100	1	8975
330	117	3001	37.4	29	+	20778105	350	3350
331	117	3679	36.4	27	+	20782443	2	3680
332	117	8420	35.3	25	+	20784845	20	8439
333	117	3133	36.6	28	+	20646354	2	3134
334	117	3314	36.9	27	+	20647327	1	3314
335	117	3476	37.2	32	+	20649420	6705	10180
336	117	3916	36.5	30	+	20719652	1	3916
337	117	3649	35.7	30	+	20720952	1441	5089
338	117	4301	37.0	31	+	20721496	2	4302
339	117	3003	34.9	23	+	20725023	494	3496
340	117	3612	36.9	26	+	20733329	1	3612

N	Unit (bp)	Length (bp)	GC%	Var%	HOR	GI	Start pos	End pos
341	117	3261	35.4	31	+	20735066	19	3279
342	118	3100	35.5	21	+	69828629	1	3100
343	118	5649	35.4	24	+	69828373	2	5650
344	118	3648	34.8	18	+	69825845	2	3649
345	118	3530	35.1	22	+	69825680	5	3534
346	118	3499	35.4	20	+	69825633	52	3550
347	118	3485	35.2	16	-	69825475	1	3485
348	118	3304	36.3	21	+	69825039	1	3304
349	118	3254	34.8	22	+	69824986	16	3269
350	118	3213	34.8	24	+	69824839	1	3213
351	118	3219	35.9	23	+	69824816	1	3219
352	118	3157	35.7	21	+	69824625	6	3162
353	118	3127	35.1	21	+	69824530	1	3127
354	118	3055	35.4	23	+	69824327	1	3055
355	118	3493	36.1	30	+	69798479	566	4058
356	118	3440	35.3	18	+	69798324	1	3440
357	118	3137	34.6	23	+	69787704	3	3139
358	118	10204	34.9	17	-	69787591	1	10204
359	118	9324	35.2	26	+	69787530	88	9411
360	118	3584	35.1	26	+	69777497	2	3585
361	174	6924	36.7	33	+	69798830	612	7535
362	174	16751	36.8	32	+	69787494	13	16763
363	175	4146	37.1	33	+	69809144	393	4538
364	175	3765	35.7	29	+	69798478	8062	11826
365	175	3989	35.9	32	+	69798311	4	3992
366	175	4225	36.2	32	+	69798309	4055	8279
367	175	6614	37.8	28	+	69779930	1	6614
368	175	3457	36.8	29	+	20668036	831	4287
369	175	4530	35.1	31	+	20734680	3450	7979
370	176	4321	34.8	28	+	69826849	8	4328
371	176	4942	36.2	27	+	69809530	767	5708
372	176	5345	36.7	30	+	69809447	1	5345
373	176	5454	35.5	35	+	69809142	2	5455
374	176	10974	36.5	30	+	69809014	1717	12690
375	176	3232	36.4	30	+	69798778	16	3247
376	176	3207	36.8	32	+	69798480	692	3898
377	176	7078	37.0	32	+	69778862	17	7094
378	176	4480	35.3	27	+	69777973	70	4549
379	176	4060	35.2	27	+	20641030	28	4087
380	176	3146	38.2	26	+	20646447	2	3147
381	176	3954	36.7	33	+	20727375	4260	8213
382	231	3409	36.2	36	+	69797997	179	3587
383	232	3239	33.5	25	+	69787692	1	3239
384	233	4151	35.2	13	-	69826637	4	4154
385	233	3853	35.1	13	-	69826172	4	3856
386	233	3449	35.9	18	+	69825474	3	3451
387	233	3073	35.1	13	-	69824357	1	3073
388	233	3008	36.0	11	-	69824084	1	3008
389	233	5022	36.4	30	+	69809020	1	5022
390	233	6244	35.5	33	+	69798831	34	6277
391	233	3077	35.6	9	-	69798155	1	3077
392	233	3948	34.3	20	+	69787329	21	3968
393	233	5237	36.2	26	+	69787007	948	6184
394	233	3849	35.7	14	-	69780432	1	3849
395	233	4339	35.9	16	-	69777996	1	4339
396	233	3515	34.7	10	+	69777424	1	3515
397	234	3397	35.9	9	-	69830835	1	3397
398	234	3636	34.3	14	-	69830656	1	3636

N	Unit (bp)	Length (bp)	GC%	Var%	HOR	GI	Start pos	End pos
399	234	6989	36.1	7	-	69829968	1	6989
400	234	3151	36.2	10	-	69829963	1	3151
401	234	7368	35.6	12	-	69829394	189	7556
402	234	7077	35.4	11	-	69829247	1	7077
403	234	4370	34.9	17	+	69829154	10	4379
404	234	3328	34.9	11	-	69829088	5	3332
405	234	6299	34.6	23	+	69828894	39	6337
406	234	5051	35.4	12	-	69828825	10	5060
407	234	6287	35.5	10	-	69828751	1	6287
408	234	4154	35.5	9	-	69828689	1	4154
409	234	5158	35.5	12	-	69828554	1	5158
410	234	5912	35.4	12	-	69828519	1	5912
411	234	5845	36.1	10	-	69828449	1	5845
412	234	5429	35.6	13	-	69828128	1	5429
413	234	4560	35.6	11	-	69828120	10	4569
414	234	4851	35.1	15	+	69827723	193	5043
415	234	4985	35.5	14	-	69827649	1	4985
416	234	4948	35.6	10	-	69827605	1	4948
417	234	4904	36.6	10	-	69827568	1	4904
418	234	4892	35.5	10	-	69827548	1	4892
419	234	3451	34.2	14	-	69827529	6	3456
420	234	4868	36.4	9	-	69827518	1	4868
421	234	4781	34.9	14	+	69827432	1	4781
422	234	4636	35.7	11	-	69827217	3	4638
423	234	4621	35.6	9	-	69827200	1	4621
424	234	4613	35.8	14	+	69827195	1	4613
425	234	4544	35.8	12	-	69827148	1	4544
426	234	4562	35.6	9	-	69827136	1	4562
427	234	4451	35.3	14	+	69827021	1	4451
428	234	4421	36.0	14	+	69826971	1	4421
429	234	4400	36.0	9	-	69826941	1	4400
430	234	4289	35.4	11	-	69826806	1	4289
431	234	4285	34.8	11	-	69826798	1	4285
432	234	4270	35.5	15	-	69826782	1	4270
433	234	4246	35.3	18	+	69826774	16	4261
434	234	4245	34.7	15	-	69826752	1	4245
435	234	4240	36.2	9	-	69826736	1	4240
436	234	4134	34.9	11	-	69826597	3	4136
437	234	4091	35.6	12	-	69826533	3	4093
438	234	4041	35.5	11	-	69826526	49	4089
439	234	4039	35.9	14	+	69826509	18	4056
440	234	4075	35.6	10	-	69826505	2	4076
441	234	4072	36.1	8	-	69826498	2	4073
442	234	4049	35.6	15	+	69826461	3	4051
443	234	3347	35.7	9	-	69826432	1	3347
444	234	3979	35.7	12	-	69826365	9	3987
445	234	3963	35.3	12	-	69826346	13	3975
446	234	3974	35.2	12	-	69826341	1	3974
447	234	3950	34.2	11	-	69826303	1	3950
448	234	3935	34.7	15	+	69826299	1	3935
449	234	3683	36.4	21	+	69826241	1	3683
450	234	3846	36.3	9	-	69826229	2	3847
451	234	3859	34.6	14	-	69826204	19	3877
452	234	3871	35.6	10	-	69826193	1	3871
453	234	3866	35.0	13	-	69826191	1	3866
454	234	3852	34.5	15	+	69826167	1	3852
455	234	3845	35.7	12	-	69826157	1	3845
456	234	3837	35.2	12	-	69826138	2	3838

N	Unit (bp)	Length (bp)	GC%	Var%	HOR	GI	Start pos	End pos
457	234	3738	35.1	13	-	69826104	60	3797
458	234	3802	35.9	7	-	69826086	9	3810
459	234	3798	35.4	12	-	69826062	1	3798
460	234	3776	35.2	9	-	69826016	1	3776
461	234	3770	34.4	14	-	69826009	1	3770
462	234	3743	35.7	15	-	69825974	3	3745
463	234	3723	35.6	9	-	69825956	1	3723
464	234	3614	34.0	16	+	69825951	105	3718
465	234	3698	36.1	10	-	69825921	3	3700
466	234	3682	36.0	8	-	69825889	3	3684
467	234	3630	34.8	18	+	69825808	3	3632
468	234	3629	35.6	9	-	69825790	1	3629
469	234	3619	35.9	12	-	69825786	8	3626
470	234	3613	35.3	16	+	69825774	1	3613
471	234	3599	36.0	12	-	69825728	1	3599
472	234	3579	35.3	10	-	69825689	2	3580
473	234	3541	35.8	10	-	69825674	32	3572
474	234	3567	35.5	12	-	69825673	4	3570
475	234	3539	35.7	14	-	69825642	8	3546
476	234	3512	35.4	13	-	69825626	1	3512
477	234	3488	35.5	16	+	69825596	1	3488
478	234	3521	35.3	9	-	69825563	1	3521
479	234	3492	35.4	9	-	69825495	6	3497
480	234	3466	35.6	9	-	69825445	5	3470
481	234	3430	35.6	17	+	69825437	34	3463
482	234	3459	35.3	11	-	69825426	1	3459
483	234	3454	34.8	10	-	69825419	1	3454
484	234	3449	35.2	10	-	69825402	1	3449
485	234	3438	35.7	10	-	69825378	1	3438
486	234	3433	36.2	9	-	69825372	3	3435
487	234	3428	35.4	11	-	69825351	1	3428
488	234	3343	35.7	11	-	69825341	1	3343
489	234	3403	34.7	18	+	69825296	4	3406
490	234	3390	35.1	14	-	69825271	1	3390
491	234	3384	36.6	4	-	69825252	1	3384
492	234	3364	36.4	6	-	69825204	1	3364
493	234	3316	36.2	8	-	69825079	1	3316
494	234	3307	34.9	11	-	69825042	1	3307
495	234	3289	35.2	9	-	69825009	1	3289
496	234	3283	36.0	7	-	69824981	1	3283
497	234	3269	35.6	9	-	69824980	1	3269
498	234	3281	36.4	9	-	69824977	1	3281
499	234	3275	35.5	9	-	69824967	1	3275
500	234	3273	35.5	9	-	69824960	1	3273
501	234	3260	35.4	14	+	69824934	1	3260
502	234	3257	35.7	10	-	69824926	2	3258
503	234	3213	36.0	7	-	69824884	30	3242
504	234	3231	36.0	8	-	69824874	10	3240
505	234	3231	35.5	12	-	69824873	4	3234
506	234	3235	35.4	10	-	69824848	1	3235
507	234	3229	35.4	9	-	69824835	1	3229
508	234	3194	35.3	10	-	69824741	2	3195
509	234	3179	35.6	11	-	69824681	1	3179
510	234	3171	35.0	11	-	69824652	1	3171
511	234	3163	35.6	13	-	69824634	1	3163
512	234	3153	36.5	11	-	69824604	1	3153
513	234	3148	35.8	10	-	69824603	1	3148
514	234	3149	36.1	7	-	69824594	1	3149

N	Unit (bp)	Length (bp)	GC%	Var%	HOR	GI	Start pos	End pos
515	234	3107	36.5	5	-	69824576	36	3142
516	234	3137	34.9	14	-	69824562	1	3137
517	234	3077	35.5	13	-	69824532	1	3077
518	234	3119	36.0	16	+	69824523	1	3119
519	234	3123	36.3	12	-	69824517	1	3123
520	234	3099	35.1	14	-	69824453	7	3105
521	234	3102	33.9	13	-	69824450	3	3104
522	234	3077	35.5	10	-	69824369	1	3077
523	234	3077	35.6	10	-	69824368	1	3077
524	234	3076	35.9	7	-	69824365	1	3076
525	234	3062	35.2	15	+	69824358	2	3063
526	234	3065	35.5	9	-	69824324	1	3065
527	234	3058	36.4	5	-	69824295	1	3058
528	234	3054	35.6	8	-	69824274	1	3054
529	234	3033	35.9	12	-	69824272	1	3033
530	234	3031	35.3	14	+	69824206	1	3031
531	234	3025	35.4	10	-	69824165	1	3025
532	234	3008	35.6	8	-	69824089	2	3009
533	234	3009	34.8	15	+	69824088	1	3009
534	234	3118	35.3	14	-	69809454	1	3118
535	234	4013	36.3	29	+	69809446	3	4015
536	234	5728	35.8	31	+	69798780	1	5728
537	234	3637	35.5	16	+	69798326	31	3667
538	234	5200	34.9	18	+	69798042	48	5247
539	234	3423	34.8	16	+	69798017	50	3472
540	234	6070	36.6	26	+	69797942	3	6072
541	234	7384	35.0	17	+	69797932	1	7384
542	234	3501	36.3	12	-	69797917	1	3501
543	234	5103	35.5	14	+	69797915	2	5104
544	234	4046	35.2	19	+	69787706	1	4046
545	234	4155	35.0	12	-	69787576	1	4155
546	234	6619	35.0	18	+	69787542	1	6619
547	234	3570	35.5	11	-	69787538	22	3591
548	234	5341	35.2	22	+	69787535	1	5341
549	234	3209	35.7	20	+	69787534	1	3209
550	234	8729	34.9	15	+	69787439	1	8729
551	234	4083	34.5	13	-	69787438	1	4083
552	234	7041	35.4	11	-	69787435	1	7041
553	234	4503	35.5	11	-	69787434	1	4503
554	234	11286	35.1	18	+	69787413	6631	17916
555	234	3385	36.4	9	-	69787373	1	3385
556	234	3556	35.7	11	-	69787371	1	3556
557	234	4579	35.7	13	-	69787180	92	4670
558	234	12819	36.1	11	-	69787179	1	12819
559	234	3862	35.0	12	-	69787069	1	3862
560	234	11702	34.6	18	+	69780671	22	11723
561	234	3099	34.5	18	+	69780488	1	3099
562	234	3239	34.9	12	-	69780288	447	3685
563	234	3433	35.0	18	+	69780252	1	3433
564	234	3790	35.6	10	-	69780160	1	3790
565	234	3048	35.1	11	-	69780047	1	3048
566	234	3085	36.1	18	+	69779902	1	3085
567	234	3438	35.9	15	-	69779900	2	3439
568	234	3536	35.0	15	-	69779405	170	3705
569	234	7952	37.4	28	+	69779404	1	7952
570	234	6279	36.2	9	-	69779250	1	6279
571	234	4356	35.4	11	+	69779249	1	4356
572	234	3672	35.7	9	-	69779059	1	3672

N	Unit (bp)	Length (bp)	GC%	Var%	HOR	GI	Start pos	End pos
573	234	16160	35.9	15	+	69778922	24	16183
574	234	4675	34.3	19	+	69778762	12	4686
575	234	5640	35.4	11	-	69778737	1	5640
576	234	4455	35.5	14	-	69778733	1	4455
577	234	3530	35.2	17	+	69778728	1	3530
578	234	3006	35.3	14	-	69778691	2	3007
579	234	5460	35.0	18	+	69778690	1	5460
580	234	5149	34.9	11	-	69778670	1	5149
581	234	4043	35.2	19	+	69778577	3	4045
582	234	7072	35.2	15	-	69778568	1	7072
583	234	3510	34.4	13	-	69778487	33	3542
584	234	4471	36.4	17	+	69778461	3	4473
585	234	3597	35.5	12	-	69778415	1	3597
586	234	3154	35.8	8	-	69778414	1	3154
587	234	4038	35.8	12	-	69778298	1	4038
588	234	3329	35.5	12	-	69778257	1	3329
589	234	3223	35.4	15	+	69778214	1	3223
590	234	4328	35.1	11	-	69778164	12	4339
591	234	4684	35.6	10	-	69778159	20	4703
592	234	5844	35.8	11	-	69778066	1	5844
593	234	4465	35.8	14	+	69778055	14	4478
594	234	4691	37.0	24	+	69778035	1	4691
595	234	4994	35.7	13	-	69778013	33	5026
596	234	3604	34.8	21	+	69777975	2	3605
597	234	3777	35.2	10	-	69777885	1	3777
598	234	3255	35.5	11	-	69777870	1	3255
599	234	7600	35.0	19	+	69777694	213	7812
600	234	4313	35.7	14	+	69777606	153	4465
601	234	3556	35.1	15	+	69777470	48	3603
602	234	3115	35.2	8	-	69777324	1	3115
603	234	3484	35.8	8	-	69777317	1	3484
604	234	4160	35.4	13	-	69777034	2	4161
605	234	4128	35.9	12	-	69777028	33	4160
606	234	3098	34.6	19	+	69776480	1	3098
607	234	3536	36.4	9	-	69775833	1	3536
608	234	3038	35.4	26	+	69591580	1	3038
609	234	3536	34.8	14	+	69970353	1	3536
610	234	3091	37.3	32	+	20778105	36	3126
611	234	3168	35.7	9	-	20778132	1	3168
612	234	9558	35.1	19	+	20787080	5	9562
613	234	5125	35.7	10	-	20787119	3	5127
614	234	3409	38.0	30	+	20668056	35	3443
615	234	4109	36.7	26	+	20716693	10	4118
616	234	3935	34.4	11	-	20731658	16	3950
617	234	3312	34.8	16	+	20741412	16	3327
618	235	4036	36.3	9	-	69826439	1	4036
619	235	3380	34.8	11	-	69825325	1	3380
620	235	3201	35.4	14	-	69825192	158	3358
621	292	6812	36.2	33	+	69809016	84	6895
622	293	3197	35.2	29	+	69824737	1	3197
623	293	5484	36.0	30	+	69787453	1	5484
624	340	4116	35.4	19	+	69798265	1	4116
625	347	3863	36.3	31	+	69787470	1	3863
626	348	3619	33.2	19	+	69787693	1	3619
627	349	3462	35.6	18	+	69825437	1	3462
628	349	3689	36.2	19	+	69778459	9	3697
629	350	3709	34.7	17	+	69825937	1	3709
630	350	7469	34.9	21	+	69779406	1	7469

N	Unit (bp)	Length (bp)	GC%	Var%	HOR	GI	Start pos	End pos
631	350	4617	35.4	17	+	69778669	1	4617
632	350	6151	34.6	19	+	69778640	4	6154
633	350	5020	35.7	20	+	69778013	1	5020
634	350	3006	35.2	15	+	20724572	1	3006
635	351	3212	35.9	18	+	69828056	2145	5356
636	351	3235	34.5	19	+	69825069	66	3300
637	351	7037	34.9	16	+	69809434	1	7037
638	351	5030	33.9	24	+	69798356	8	5037
639	351	3536	34.8	23	+	69787333	1	3536
640	351	4043	34.7	19	+	69778399	1	4043
641	351	3355	34.5	14	-	20646358	1	3355
642	352	3651	34.8	20	+	69825845	5	3655
643	352	3356	34.4	13	-	69825186	1	3356
644	406	4778	35.0	25	+	69809541	774	5551
645	416	4567	35.0	24	+	69809541	1075	5641
646	466	3506	35.0	13	-	69825607	31	3536
647	466	3434	36.2	10	-	69825365	1	3434
648	466	3173	36.3	13	-	20673162	1	3173
649	467	9869	35.4	17	+	69830098	20	9888
650	467	5725	35.6	9	-	69828626	351	6075
651	467	5183	35.1	9	-	69827882	2	5184
652	467	4378	35.5	12	-	69826947	28	4405
653	467	3816	35.2	9	-	69826095	1	3816
654	467	3459	35.5	14	-	69825542	3	3461
655	467	3265	36.3	12	-	69824944	1	3265
656	467	3024	34.8	13	-	69824305	37	3060
657	467	3335	34.8	14	-	69797936	15	3349
658	467	5369	35.9	12	-	69787382	8	5376
659	467	3272	35.2	9	-	69778215	3	3274
660	467	3068	36.1	9	-	69777345	11	3078
661	467	3248	34.5	14	+	69777259	11	3258
662	467	3137	34.8	16	+	20736854	1	3137
663	467	11354	35.8	12	-	20746950	1	11354
664	468	3258	35.8	13	-	69824963	1	3258
665	468	3556	34.8	13	-	69798151	38	3593
666	468	4156	35.8	12	-	20643713	2	4157
667	469	3023	34.3	21	+	69778761	1	3023
668	469	3096	35.8	10	-	69778084	535	3630
669	471	3297	36.2	30	+	69798480	10622	13918
670	522	4387	36.7	28	+	69797955	186	4572
671	522	6561	36.4	31	+	69787468	454	7014
672	583	3934	34.3	17	+	69787329	47	3980
673	583	3977	35.4	18	+	69777573	1	3977
674	584	4040	34.2	18	+	69826447	1	4040
675	585	3130	35.0	14	+	69824551	3	3132
676	586	3448	35.2	17	+	69825399	1	3448
677	641	3736	35.8	22	+	69826026	11	3746
678	701	3731	35.5	21	+	69825999	1	3731
679	701	3746	35.8	19	+	69825989	7	3752
680	701	3606	35.4	14	+	69825841	1	3606
681	701	3404	35.8	16	+	69825679	163	3566
682	701	3314	35.8	13	-	69825072	1	3314
683	701	4077	35.8	9	-	69787383	1	4077
684	703	4558	34.9	16	+	69798358	6	4563
685	703	3328	36.1	13	-	69777607	1	3328
686	703	5037	35.3	10	-	20745496	1	5037
687	819	4049	35.6	19	+	69826533	46	4094
688	821	3564	34.8	7	-	69825657	1	3564

N	Unit (bp)	Length (bp)	GC%	Var%	HOR	GI	Start pos	End pos
689	821	3257	35.0	8	-	69825052	1	3257
690	927	4759	34.6	20	+	69798268	1	4759
691	932	3225	34.6	21	+	69824825	1	3225
692	933	3012	35.0	10	-	69824127	1	3012
693	933	4014	35.2	16	+	69780289	1	4014
694	935	3448	35.4	9	-	69825400	1	3448
695	935	4985	35.8	9	-	69780372	1	4985
696	935	3803	35.9	9	-	69777344	1	3803
697	981	4918	34.2	25	+	69778183	255	5172
698	1052	3565	35.4	13	-	69825673	1	3565
699	1055	3824	35.0	16	+	69787541	1	3824
700	1056	3764	35.6	13	-	69826625	2	3765
701	1168	3267	35.7	20	+	69780673	1	3267
702	1227	4530	34.9	18	+	20739849	103	4632
703	1277	3214	35.9	13	-	69828056	2146	5359
704	1284	3576	35.9	13	-	69825693	7	3582
705	1288	3406	34.9	13	-	69798017	1	3406
706	1343	4203	36.2	12	-	69828297	41	4243
707	1395	8369	36.2	21	+	69797957	5728	14096
708	1396	3844	35.3	8	-	69829461	146	3989
709	1403	3815	36.1	9	-	69826105	6	3820
710	1443	5271	37.3	19	+	69809461	1	5271
711	1518	4626	34.7	13	-	69827227	17	4642
712	1521	4205	35.8	12	-	69826694	2	4206
713	1634	8320	36.4	10	-	69779065	1	8320
714	1737	3551	35.8	11	-	69798832	1	3551
715	1756	3887	35.2	8	-	69826226	7	3893
