# Supplementary material for: Tandemly repeated DNA families in the mouse genome
Source: BMC Genomics. 2011 Oct 28;12:531. doi: 10.1186/1471-2164-12-531 (PMC3218096; doi:10.1186/1471-2164-12-531)
Supplement: Additional file 3 — Supplementary figures. This file can be viewed with: Adobe Acrobat Reader. [file 1471-2164-12-531-S3.PDF]

Additional file 3 – Figures S1 and S2

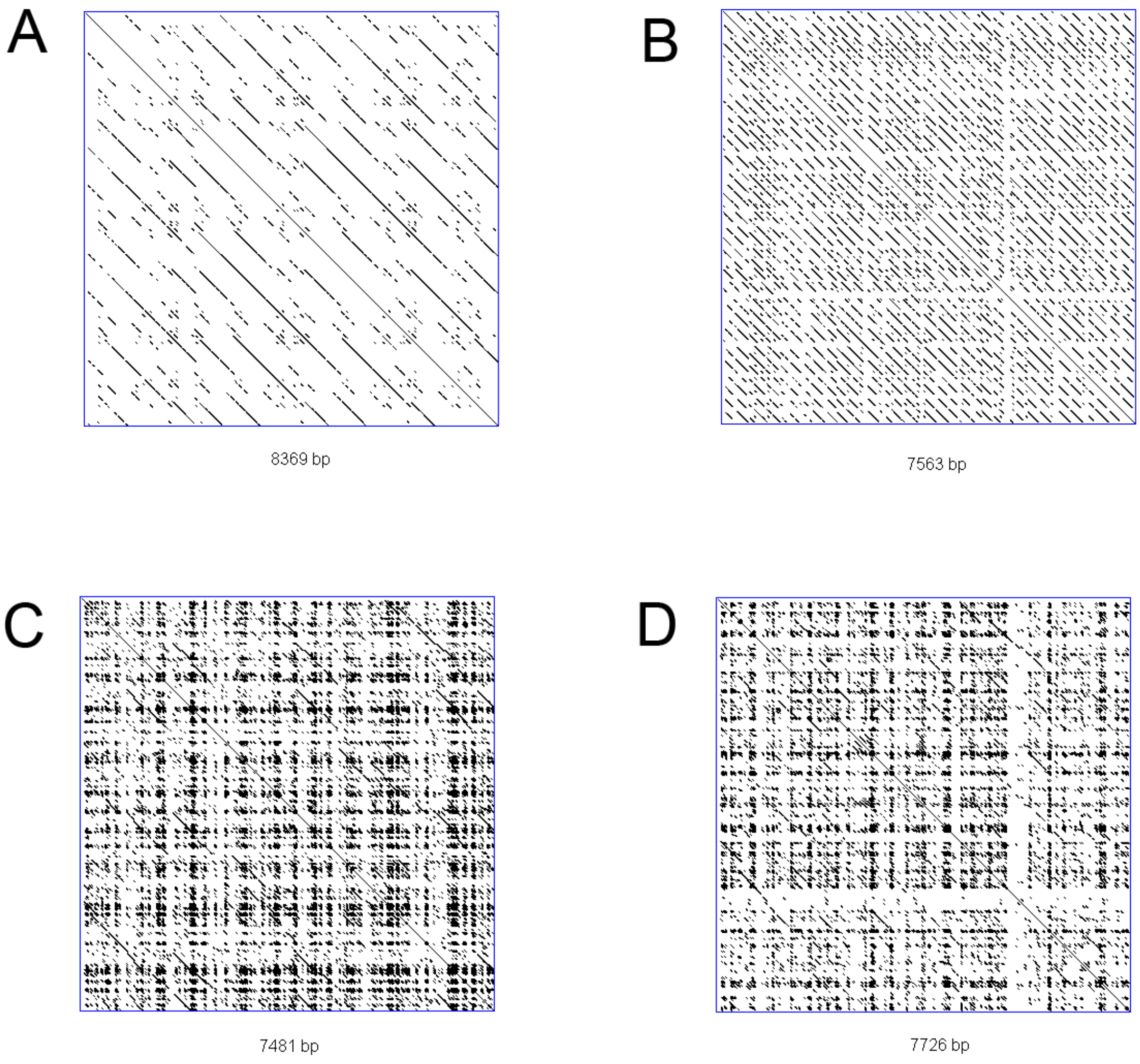

Figure S1 – The dot-plot similarity analysis of MaSat and TRPC-21A-MM
The black and white color dot-plot was performed with a window size of 51 bp and minimum identity of 90% for MaSat and 80% for TRPC-21A-MM. A – the dot plot of the MaSat array N707 (Additional file 2) same as on Figure 4; B - MaSat array N4 (Additional file 2); C - TRPC-21A-MM array N50 (Additional file 1, Table S3); D - TRPC-21A-MM array N8 (Additional file 1, Table S3).

With different window size it is possible to notice different features in HOR structure of tandemly repeated DNA. The similarity visualization using 13 bp window size and full grey scale can be used for distinguishing large HORs of about 2 kb (Figures 4A, 5A) as well as little units of less than 20 bp at high magnification (Figures 4C, 5C). The similarity visualization using 51 bp window size and two colors allow to notice the overall difference between different MaSat

arrays (Additional file 2 and Figure 3). The existence of very large HORs is visible in such a case and HOR of about 1 kb is more obvious for both TR.

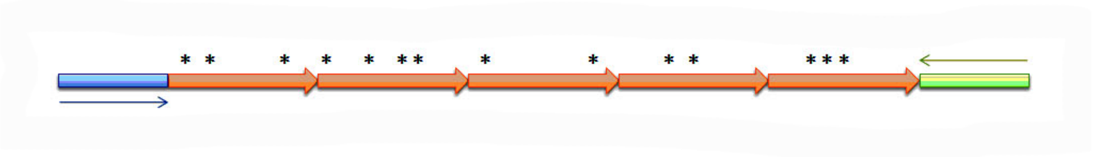

Figure S2 - Double stranded FISH probes design.
Array-specific synthetic oligonucleotides were designed for FISH. Four TRPC-21A-MM repeated monomers from the most specific region of the array was chosen (orange) and flanked by two adapters (blue, green). To amplify and label probe two primes were used (arrows). The monomer variability is shown by asterisks.
